# Interactions between Humans and Dogs during the COVID-19 Pandemic: Recent Updates and Future Perspectives

**DOI:** 10.3390/ani13030524

**Published:** 2023-02-02

**Authors:** Mohamed S. Kamel, Amr A. El-Sayed, Rachel A. Munds, Mohit S. Verma

**Affiliations:** 1 Department of Agricultural and Biological Engineering, Purdue University, West Lafayette, IN 47907, USA; 2 Department of Medicine and Infectious Diseases, Faculty of Veterinary Medicine, Cairo University, Giza 12211, Egypt; 3 Krishi Inc., West Lafayette, IN 47906, USA; 4 Weldon School of Biomedical Engineering, Purdue University, West Lafayette, IN 47907, USA; 5 Birck Nanotechnology Center, Purdue University, West Lafayette, IN 47907, USA

**Keywords:** SARS-CoV-2, diagnosis, COVID-19, companion, pets, bio-detection, social distancing, infection, epidemiology, ACE2

## Abstract

**Simple Summary:**

Dogs are considered man’s best friend, with many owners sharing a close relationship with their dogs. This affection increased during the COVID-19 pandemic, as owners spent more time with their canines during the lockdowns and quarantines. During this time, owners noted how their dogs helped reduce their stress and anxiety. Yet, of concern is the known natural infection/transmission of SARS-CoV-2 reported in numerous animals (e.g., cats, minks, deer), necessitating the need to determine if dogs can contract or spread SARS-CoV-2. In this review, we focused on the human–canine interface of COVID-19 and the positives and negatives of this interface. We found that dogs can get COVID-19 from their owners but are asymptomatic when infected—it is unclear if it affects the health of dogs. Research is inconclusive on if dogs can transmit SARS-CoV-2 to their owners. Although dogs could be hosts, they are beneficial beyond just stress relief. Sniffer dogs can rapidly detect COVID-19-positive individuals in crowds, with almost 80% success in distinguishing between positive and negative people. Overall, we emphasize that more research is needed to understand the role of dogs in the interspecies spread and even contraction of SARS-CoV-2.

**Abstract:**

COVID-19 is one of the deadliest epidemics. This pandemic is caused by severe acute respiratory syndrome coronavirus 2 (SARS-CoV-2), but the role of dogs in spreading the disease in human society is poorly understood. This review sheds light on the limited susceptibility of dogs to COVID-19 infections which is likely attributed to the relatively low levels of angiotensin-converting enzyme 2 (ACE2) in the respiratory tract and the phylogenetic distance of ACE2 in dogs from the human ACE2 receptor. The low levels of ACE2 affect the binding affinity between spike and ACE2 proteins resulting in it being uncommon for dogs to spread the disease. To demonstrate the role of dogs in spreading COVID-19, we reviewed the epidemiological studies and prevalence of SARS-CoV-2 in dogs. Additionally, we discussed the use of detection dogs as a rapid and reliable method for effectively discriminating between SARS-CoV-2 infected and non-infected individuals using different types of samples (secretions, saliva, and sweat). We considered the available information on COVID-19 in the human–dog interfaces involving the possibility of transmission of COVID-19 to dogs by infected individuals and vice versa, the human–dog behavior changes, and the importance of preventive measures because the risk of transmission by domestic dogs remains a concern.

## 1. Introduction

Severe acute respiratory syndrome coronavirus 2 (SARS-CoV-2), which emerged in Wuhan, China, in 2019, is the etiological agent of the COVID-19 pandemic. It has spread to over 600 million people worldwide, with a death toll of more than 6.5 million as of October 2022. COVID-19 has become one of the most important economic, health, and humanitarian problems of the 21st century.

The outbreak of SARS-CoV-2 has prompted intensive research into its epidemiological aspects in both humans and animals. To understand its transmission dynamics, risk factors, and clinical characteristics, studies have been carried out in both populations. SARS-CoV-2 is primarily spread through respiratory droplets when an infected individual sneezes, talks, or coughs [1,2]. There has been speculation that the virus is present on surfaces or objects and can spread through aerosol transmission in specific situations [3]. This virus is highly contagious, with its basic reproductive number (R0) estimated to range between two and three [4]. Research has indicated multiple potential risk factors for serious illness and death from COVID-19 in humans, most notably older age and comorbid conditions such as obesity, diabetes, and cardiovascular disease [1]. Manifestations of the virus vary drastically, from asymptomatic to deadly or life-threatening conditions. Common symptoms include fever, cough, shortness of breath, and fatigue, with anosmia and ageusia reported in some cases. In extreme circumstances, COVID-19 led to pneumonia, acute respiratory distress syndrome (ARDS), and organ failure [1,2].

In addition to the major COVID-19 outbreaks, hospitalizations, and deaths in humans, there were initial reports of the disease in a number of animal hosts (cats, tigers, dogs, minks, deer, and lions) [5,6,7,8,9,10,11,12,13,14,15]. Currently, numerous species have been identified and reported to be infected with SARS-CoV-2, including raccoons, hamsters, ferrets, coatimundi, fishing cats, hippopotamuses, snow leopards, pangolins, gorillas, hyenas, otters, rabbits, puma, armadillo, red fox, coati, cattle, Eurasian beaver, binturong, lynx, leopard, manatee, black-tailed marmoset, giant anteater, squirrel monkey, and mandrill. Such findings provide valuable insight into the transmission of SARS-CoV-2 across the animal kingdom [5,6,7,8,9,10,11,12,13,14,16,17,18,19,20,21], as well as highlighting the problem of zoonotic diseases, i.e., diseases that can spread from animals to humans. It is thought that the virus jumped from bats to humans through an intermediate host such as a pangolin [22,23]. Recent studies have revealed the ability of SARS-CoV-2 to move from one host to another, such as being able to proliferate in ferrets, which are commonly used as animal models to study human respiratory viruses, as well as infect cats, dogs, and a number of non-human primates [1,2,3,4,5,6,7,8,9,10]. This raises concerns about the possibility of pet animals to serve as a reservoir for the virus. The overwhelming majority of infectious diseases in humans are of animal origin, with roughly 75% of them being zoonotic [24,25]. This draws attention to the need for more robust surveillance, coupled with intensive research, in order to better understand how zoonotic illnesses can affect human health. Moreover, certain factors, such as the destruction of natural habitats and the close proximity of wildlife, livestock, and people, all contribute to the emergence of zoonotic diseases. This is especially poignant in the case of SARS-CoV-2, where animal markets and the wildlife trade played a critical role in the virus’ spread and emergence. Consequently, a One Health approach, which unites human, animal, and environmental health, and bears in mind their mutual dependence, is imperative in combating zoonotic diseases [26,27]. This approach highlights the necessity of cooperation among human healthcare professionals, veterinarians, and environmental scientists to pinpoint and lessen the chances of new disease emergences. This should involve recognizing the essential host species and intermediate host species participating in disease transmission and examining the virus’ genetic makeup and its capacity to adjust to new host species. Moreover, research should be conducted to upgrade our comprehension of the mechanisms of zoonotic disease emergence and transmission.

One way to track disease emergencies and outbreaks is through geographic information systems (GIS), which provide a powerful means for evaluating and representing partial data across many sectors, including veterinary science [28]. By enabling the integration, control, and analysis of different types of data, such as population distribution, clinic localities, and disease outbreak records, GIS technology has become a vital instrument for veterinary professionals, researchers, and policymakers in their attempts to tackle multifaceted problems related to animal health and welfare [29,30]. In the veterinary sector, a primary application of GIS has been in the field of disease monitoring and outbreak. GIS technology has been instrumental in addressing outbreaks through its capacity to compile, store, and analyze vast spatial data on the progression, position, and seriousness of epidemics [29,31]. GIS technologies can be used to identify high-risk areas, track the spread of diseases, and develop strategies for controlling and preventing further outbreaks. For instance, GIS can assist in recognizing areas where the risk of disease transmission is elevated and help devise targeted control measures to be implemented. Additionally, GIS can be employed to map the locations of veterinary clinics, animal hospitals, and other animal health services and resources. These data can be deployed to discern regions that lack adequate veterinary services and create plans to expand access to animal health care during normal times and during pandemics [32]. On top of that, GIS can be utilized for plotting the positions of animal feed and supply stores, aside from other necessary resources for animal health and welfare [29,33]. GIS technology can be employed in animal welfare research and management. It can be utilized to plot the location of animal welfare facilities, such as shelters and sanctuaries, and to analyze the factors that affect animal well-being and the impact of the pandemic on them [34]. Additionally, GIS can record and examine the movements of animals/wild animals to detect potential hazards to animal populations, such as habitat destruction [34,35]. Furthermore, GIS is used in veterinary epidemiology, which is the study of the distribution and causes of animal diseases [36]. GIS can be employed to map the propagation of diseases, recognize risk factors, and develop disease control techniques [29,37]. It can also be used to monitor the spread of diseases and to assess the efficiency of disease control efforts. To sum up, GIS technology has become a critical instrument for veterinary experts, researchers, and decision-makers trying to comprehend and tackle complicated matters related to animal health and welfare [29,33,38]. The capability to assess and visualize spatial data facilitates the construction of more proficient and precisely tailored tactics for disease observation, outbreak management, and animal well-being [35]. As the technology advances, the potential utilization of GIS in the veterinary sector will continue to proliferate and play an ever more critical role in deconstructing the troubles confronting animal health and welfare. In addition to GIS as a tool to map and trace the spread and distribution of infectious diseases, loop-mediated isothermal amplification (LAMP) assay has been established as a swift, straightforward, and cost-effective molecular technique that can be employed as a point-of-care surveillance tool for infectious diseases in both human and animal populations [39,40,41,42,43,44,45,46]. Its notable sensitivity, specificity, and capacity to detect minimal levels of pathogenic DNA/RNA render it an invaluable tool to monitor and control the transmission of infectious diseases, particularly in scenarios that necessitate speedy results [40,41,42,43,44,45,46,47].

Genetics and genetic evolution help to improve our understanding of the evolution of the virus, which helps in applying novel technologies, like GIS. Using whole genome sequencing (WGS) data, SARS-CoV-2 is classified as a β-coronavirus of the subgenus Sarbecovirus within the family Coronaviridae. Its RNA genome (approximately 30 kb) encodes four structural proteins and 16 non-structural proteins [48]. The structural proteins are membrane (M), spike (S), nucleocapsid (N), and envelope (E) [49]. The S protein of coronaviruses resembles a crown that forms a spike on its surface and is critical to host receptor recognition, host range determination, viral tissue tropism, binding, entry, fusion, and induction of T cell response and neutralizing antibodies [50,51]. The specific form of the viral envelope is formed by the M protein, which also mediates inflammatory responses and forms ribonucleoproteins. While the N protein supports vital entry and survival in host cells, the E protein enhances pathogenicity and acts as an ion channel [50]. Owing to its high mutation rate, SARS-CoV-2 acclimates to a wide host range [52].

In light of conflicting reports regarding the role of domestic animals in the emergence of the pandemic, there has been growing concern among pet owners. For this reason, further research is needed to better understand the dynamics of the disease, particularly with regard to the ability of the virus to jump species and be transmitted from people to animals and conversely. Through a literature review of papers published in scientific journals, this study highlights reported cases of COVID-19 infection, susceptibility, and spread in dogs and focuses on different aspects of the human–dog interface during the COVID-19 pandemic. Despite the numerous cases of SARS-CoV-2 transmission, caution and additional research are needed to better understand the dynamics of disease transmission through the environment and between species. Future human–animal research can contribute to the development and enforcement of measures to prevent further COVID-19 transmission. This review covers the present scenario and anticipated outlook of COVID-19 in dogs.

## 2. Human–Canine Interface: SARS-CoV-2 Infection, Susceptibility, Epidemiology, and Transmissibility in Dogs

### 2.1. SARS-CoV-2 Infection of Dogs

In 2018, over 470 million dogs were owned worldwide [53], with the highest number of dog owners in the United States, China, and Russia. Initial reports of canine susceptibility to SARS-CoV-2 were published in Hong Kong, where 2 of 27 dogs tested positive [54]. As of February 27, 2020, the first dog had been diagnosed [17]. A Pomeranian was identified as SARS-CoV-2 RNA positive via a nasal and oral cavity swab. The owner was also infected with the virus, implying a possible transmission from human to dog. The viral titers in the dog samples were very low, and there were no clinical signs, but a genetic analysis showed a close association between canine and human viruses. A few days later, blood tests revealed the presence of neutralizing antibodies. After being quarantined for three days, the dog passed away due to unrelated health reasons, not from SARS-CoV-2 [54,55]. On 18 March 2020, a second dog tested positive [54]. Several weeks after testing positive for SARS-CoV-2 RNA, a 2.5-year-old German shepherd developed neutralizing antibodies. Presumably, the dog was infected by the COVID-19-positive owner [54,55].

However, most data pointed to the low susceptibility of dogs to SARS-CoV-2, especially to the early viral variants, as monitored dogs did not show infection or clinical signs [56]. An earlier report from France described an absence of SARS-CoV-2 infections among dogs exposed to COVID-19 patients at a veterinary campus [57]. In fact, none of 21 pets (9 cats and 12 dogs) showed signs of infection, even though they lived in close contact with their owners (including a group of 20 veterinary students, 2 of whom tested positive for COVID-19) who showed clinical signs of infection (fever, anosmia, cough,). Based on molecular PCR tests or immunoprecipitation assay, no detection of SARS-CoV-2 RNA or antibodies was found in the pet’s blood 30 days following the index case [57]. Similarly, two dogs whose owners were hospitalized for COVID-19 were quarantined from 18–20 March 2020. One of these dogs was diagnosed as positive for SARS-CoV-2, and the virus could be recovered from it. However, throughout the quarantine duration, no clinical signs were seen [58,59], highlighting that even though dogs may have tested positive for SARS-CoV-2, this dog did not display obvious signs of infection (e.g., coughing, fever). Similarly, in early 2020, no viral RNA was found in 12 dogs residing with people reported to be infected with COVID-19 in northern Spain [60]. These data show that dogs did not have a pronounced role at the beginning of the pandemic.

Examples of human-to-animal transmission indicate that mainly cats show clinical signs, with rare signs in dogs [61]. Although dogs and cats are affected by SARS-CoV-2, it remains uncertain whether they can transmit the virus to humans. Within domestic animals, infection is possible in ferrets and cats, but unlikely in dogs, pigs, and chickens [62]. Cats have become infected by respiratory droplets from other animals, including rom humans [63,64,65]. Dogs can be infected experimentally and when infected show signs of fever, weight loss, and viral shedding via feces, nasal secretions, and urine [64]. Furthermore, lung tissue from the experimentally infected dogs showed mild interstitial pneumonia, and elevated lactate dehydrogenase levels [64].

Of concern is that the new mutations of SARS-CoV-2 appear to be more pathogenic to dogs. Currently, there are almost 70 diagnosed cases of dogs (diagnosed by reverse transcription-quantitative polymerase chain reaction (RT-qPCR)) worldwide [66]. SARS-CoV-2-positive dogs have been found in Croatia, Thailand, Brazil, Italy, USA, Mexico, Japan, Argentina, Germany, Hong Kong, and Bosnia and Herzegovina [56]. SARS-CoV-2 antibodies were identified in 1.21% of Thai dogs and 3.3% of Italian dogs [67]. Similarly, anti-SARS-CoV-2 neutralizing antibodies were found in the serum of stray dogs in Rio de Janeiro, Brazil [68]. And in France, a longer-term study found nasal swabs from a dog living with infected owners were positive for SARS-CoV-2 using RT-qPCR. The swab remained positive one month after the COVID-19 diagnosis, with the first appearance of anti-SARS-CoV-2 antibodies being detected by enzyme-linked immunosorbent assay (ELISA) on day 12. The antibodies persisted for five months. Through WGS, the study found that the viral isolates of the dog showed 99% and 100% identity with the sequences of the owner and his wife, respectively [56].

Due to the conflicting data, it is unclear whether the most recent variants became more infectious to dogs compared to older strains, specific lineages of the virus evolved in parallel in animals, or diagnostic testing in dogs improved. The extent to which dogs contribute to the epidemiology of the disease is currently unknown [56]. However, it was noted that dogs infected with the earlier variants did not exhibit clinical signs. However, in the case of the Marseille-4 or B.1.160 variant, the susceptibility of dogs to the disease increased, and infected dogs began to develop mild clinical signs such as rhinitis [56]. Later, infection with other newer viral mutants, such as the British B.1.1.7 variant, led to the appearance of atypical clinical manifestations, which include clinical signs such as myocarditis associated with severe cardiac abnormalities in dogs and cats [69,70]. However, the B.1.1.7 variant infection had also been reported with only sneezing [57], or even asymptomatic presentation [71]. Infection with SARS-CoV-2 B.1.177 variant also impacted the canine digestive system, leading to haemorrhagic diarrhea [72].

### 2.2. Dog Susceptibility to Infections and Its Link to Receptors

Animals are infected with SARS-CoV-2 through the intra-nasal route via the angiotensin-converting enzyme 2 (ACE2) [73], a receptor for cell entry [74,75,76]. The presence of antibodies against SARS-CoV-2 has been reported as a result of seroconversion in dogs. This seroconversion is an indication of weak canine infection leading to an immune response [77]. Because of the poor virus replication caused by the low number of ACE2 receptors in dogs, no severe clinical signs are expected in most cases and the most important clinical sign in infected dogs is rhinitis [56].

**Figure 1:**
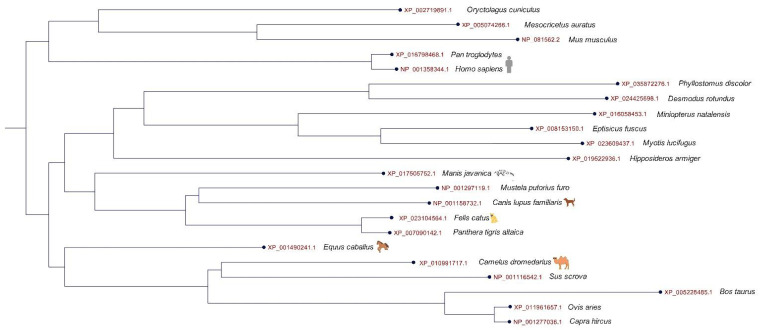
Phylogram of the angiotensin-converting enzyme 2 (ACE2) receptor amino acid sequences in humans and different animals including dogs, cats, pangolin. The ACE2 amino acid sequences were downloaded from NCBI, and the tree was generated using a maximum likelihood estimate under a JTT model and with a bootstrap 1.000. The phylogenetic tree was constructed using MEGA X software.

An important function in COVID-19 pathogenesis has been demonstrated for the host receptor code for ACE2, which determines the specificity and range of interaction between the virus and the receptor [78]. The amino acid parts of the ACE2 receptor of different organisms indicate the phylogenetic distance to the human ACE2 receptor, and show the ACE2 receptor sequences of pangolins, cats, and dogs are close to each other (Figure 1). Additionally, the ACE2 receptor in these animals can be used to predict the SARS-CoV-2 S protein in domestic cats, hamsters, pigs, dogs, as well as other species [58]. In addition to the human ACE2, the spike of SARS-CoV-2 shows extensive tropism for ACE2 in dogs, cats, and cattle [79] suggesting the susceptibility of dogs to SARS-CoV-2 infection.

In some dogs, seroconversion with antibody responses occurred but not from active infections. The lower vulnerability of dogs to SARS-CoV-2 compared to other domestic animals, such as cats, is mainly due to the differences in the structure of their ACE2 gene and the SARS-CoV-2 spike protein receptor binding domain (RBD) [80]. Residues that interact with ACE2 (amino acids 27, 31, 34, and 82) have been shown to affect the receptor specificity of SARS-CoV-2 [81]. Moreover, even single substitutions of amino acids such as H34Y in dogs have important effects on ACE2 associated with the RBD spike SARS-CoV-2 protein, even though ACE2 has largely conserved amino acid sequences. The H34Y mutation in dogs has been found in other vertebrates, including primates, and suggests an alteration that interacts with the RBD spike protein which disrupts Y453 hydrogen bonding, resulting in reduced susceptibility to the virus [82]. Such a mutation might explain the differential susceptibility of cats versus dogs to SARS-CoV-2 infection since cats lack this H34Y mutation [80].

Although possible pathways have been identified to explain why cats are more susceptible to SARS-CoV-2 infection, an in-depth understanding of the zoonotic potential of pet-borne coronaviruses and their role in outbreak surveillance requires further investigations, particularly to understand if such pathways exist in our canine companions. However, given the possibility of contact between pets and humans, caution must be exercised when pets interact with people more prone to contracting COVID-19, such as older adults that may be immunocompromised. Additionally, the classification of the actual SARS-CoV-2 species of origin is still debated. Collective data and analyses suggest that the power of evolutionary molecular biology may play a role in the recognition of biochemically testable theories of host–pathogen interactions (key lock mechanism) and zoonotic transmission potential, aiding in determining the initial reservoir and possible future reservoirs of concern.

Cross-species residual identifications of such interactive residues are listed in the ACE2 range (32–100%) [78,83,84]. Moreover, many short isoforms of ACE2 in domestic animals, including pigs, cattle, and dogs, exhibit N-terminal truncation of 10–13 major residues in the spike-RBD binding network but retain enzyme activity [85]. For instance, Shi et al. reported that SARS-CoV-2 is capable of replicating in chickens, pigs, ducks, and dogs, but its replication is poor [65,86]. In addition, interspecies comparisons show that human and cat ACE2 proteins are more similar to each other than those of dogs, ferrets, mice, and rodents, with the binding affinity of spike and ACE2 proteins differing significantly from these animals in comparison to humans [87]. Moreover, ACE2 proteins act as receptors for the virus entry in dogs, pigs, and even sheep and cattle. The apparent lower infection risk of these species might be related to the noted relatively lower respiratory tract ACE2 content [88]. Furthermore, the immunohistochemistry revealed immunolabeling of ACE2 to be present only in the lung endothelium and tunica media but not in the respiratory tract of these animals [89].

**Figure 2:**
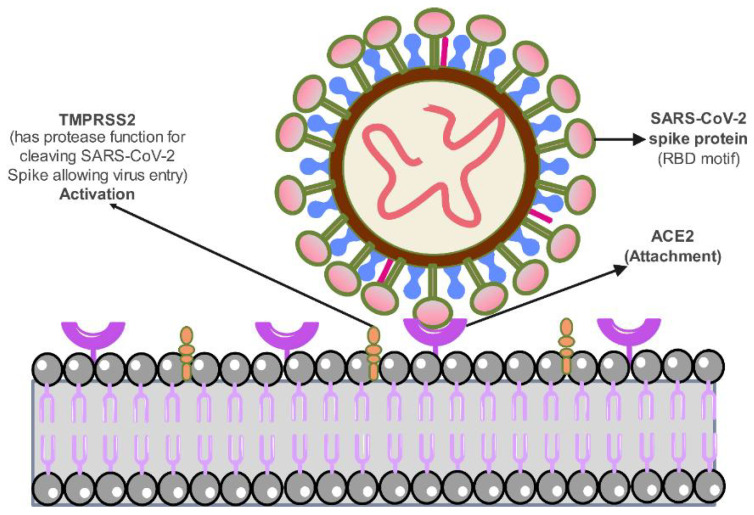
Schematic illustration displays the role of ACE2 and TMPRSS-2 receptors in SARS-CoV-2 attachment and infection.

As previously stated, the canine susceptibility to SARS-CoV-2 infection is restricted, at least for older variants. Moreover, studies on experimentally infected dogs are inconclusive, with some showing asymptomatic infections with no clinical signs, no replication or transmission to contact animals, and no pathological changes at necropsy, resulting only in an antibody response, and others showing the converse, as previously discussed [53,64]. For example, five beagles were intranasally infected with SARS-CoV-2, and samples were collected over the following two weeks [65]. Between 2–6 days, SARS-CoV-2 viral RNA was post-infection (dpi) in three of the dogs from rectal swabs. Yet, during this testing period, virus isolation or virus detection in dogs remained negative based on oropharyngeal swabs or tissue autopsies—explaining the inconsistencies in studies. In addition, Chen et al. (2020) reported very low co-expression of ACE2 and transmembrane serine protease 2 (TMPRSS-2) target receptors in canine lung cells and mutations which are presumably responsible for the low receptors of the essential amino acid sequences in ACE2 receptors [90]. To improve understanding, the roles of ACE2 and TMPRSS-2 in the SARS-CoV-2 virus entry are illustrated in Figure 2.

### 2.3. Epidemiological Prevalence of SARS-CoV-2 Infection in Dogs: Molecular and Serological Surveys

Dogs and cats can be infected with alphacoronaviruses and betacoronaviruses (Canine CoVs, and Feline CoVs) [91], and are susceptible to SARS-CoV-2 infection. Even though canines are susceptible to coronavirus infections, such incidences are low throughout Europe [92]. However, the biological, epidemiological, and virological characteristics of coronaviruses, focusing primarily on spike protein plasticity, imply the possibility of cross-species transmission—sick pet owners can get their pets sick [93,94,95]. This risk is emphasized by the observation of numerous cases of infections in cats and dogs which reported infection rates as high as 15.8% [54,96,97]. Despite these findings, reports continue to suggest that pet owners are not at risk of infection and companion animals play an insignificant role in the spread of the disease. Yet, newer studies focusing on the seroprevalence of SARS-CoV-2 antibodies in pets if finding strong links to an animal being seropositive and living in a COVID-19+ household. These seropositive studies found that 53% of pets in COVID-19+ households had antibody responses to SARS-CoV-2. Furthermore, many of the study pets lived in single-pet households, implying transmission was not from pet to pet but from owner to pet. Such findings highlight human-to-animal transmission and raise the question if pets are capable of transmitting SARS-CoV-2 to their owners [98]; studies exploring the abilities of companion animals to infect their owners are limited.

In a recent longitudinal study conducted in Texas, USA, researchers examined dogs and cats under natural infection in a household with COVID-19-infected patients. They found 15.3% of 59 dogs were positive for SARS-CoV-2 based on neutralizing antibodies or RT-PCR and genome sequencing. In addition, living in a COVID-19-contaminated environment was a possible cause for two PCR COVID-19-positive dogs that were tested with rectal and body swabs without detection of neutralizing antibodies on resampling [99]. A PCR COVID-19-positive dog showed an increase in virus-neutralizing antibodies at different visits over time [99]. Additionally, in a large-scale study in Italy, no dogs tested positive for COVID-19 using PCR, however, 3.3% (15/451) of dogs showed seroconversion for SARS-CoV-2, and dogs living with SARS-CoV-2-infected patients were significantly more likely to be positive for COVID-19 than the COVID-19-negative households [100]. Furthermore, studies are even finding SARS-CoV-2 infection in free-ranging dogs in a rural indigenous community in the Ecuadorian Amazon in oral and nasal swab samples [101]. All these findings emphasize how prevalent COVID-19 is in dogs, and the need to incorporate a variety of testing measures to detect their susceptibility more accurately to the virus.

Even though the evidence is limited on the ability of companion animals to transmit COVID-19 to humans, these newer studies have led authorities in Hunan and Zhejiang provinces in China to kill animals found in public places as a measure to avoid virus transmission. In Hong Kong, dogs were tested for the presence of SARS-CoV-2. It is important to reduce the spread of COVID-19, but more research is needed to accurately understand the risk of animal-to-animal and animal-to-human transmission, and the route of infection (especially the duration of shedding and viral load) under natural conditions. To determine the frequency of pet infection, the identification of risk factors for infected animals, and the impact of pet infection on COVID-19 epidemiology, a comprehensive, longitudinal serological analysis of SARS-CoV-2 infection in pets is needed.

To address this gap, infections with SARS-CoV-2 were screened from June to September 2021 among domestic pets (n = 225) in Bangkok and the surrounding area in Thailand and confirmed for one dog and one cat belonging to COVID-19-positive households. An analysis of WGS data identified a delta variant of SARS-CoV-2 called B.1.617.2, and a phylogenetic analysis showed that the SARS-CoV-2 isolated from canines and felines belonged to the AY.30 and AY.85 sub-lineages, respectively. After the detection of viral RNA, anti-SARS-CoV-2 antibodies were confirmed in both the dog (day 9) and the cat (day 14). In light of the human–animal interface, this study emphasizes the danger of the variant of concern spreading to companion animals. Therefore, monitoring SARS-CoV-2 in companion animals is needed on a routine basis [102]. In addition, indirect ELISA revealed that 16 of 946 canine sera sampled during the outbreak, including 36 obtained before the pandemic outbreak, were positive for the SARS-CoV-2 RBD. There were detectable SARS-CoV-2-specific neutralizing antibodies in 10 of these 16 sera samples, with titers ranging from 1/20 to 1/180. It is interesting to note that the number of SARS-CoV-2 seropositive dogs decreased when the outbreak was effectively controlled, which demonstrates the possible transmission of COVID-19 from owners to their pets [103]. Other studies exploring human–animal transmission found sharing food with an infected owner was statistically significantly associated with SARS-CoV-2 positivity in companion animals. Additionally, although viral shedding from animals is insufficient to be transmitted to animals or humans during walks, precautions should also be taken for close contact as part of the global health strategy. Surveillance of SARS-CoV-2 in pets is essential for determining the impact of pet–human contact on COVID-19 transmission, as free-roaming cats and dogs are common worldwide, and coronaviruses have great potential for interspecies transmission [104]. A more complete guide of known COVID-19 studies in dogs has been presented in Table 1.

### 2.4. COVID-19 Transmissibility between Companion Animals and Humans

Bats or pangolins have been considered the source of initial SARS-CoV-2 transmission to humans [26,27,110,111]. It has been shown that SARS-CoV-2, like other coronaviruses, infects various species of animals, including ferrets, cats, and dogs, though the viral loads of these animals are relatively low. Therefore, there are some cases in which domestic animals have contracted SARS-CoV-2, although the primary mode of transmission is human-to-human [112]. Although animal-to-human SARS-CoV-2 transmission in companion animals (dogs, cats) has not been documented, it is possible that pet ownership could expose the most vulnerable population at risk: older adult pet owners. Epidemiological evidence indicates that more severe etiopathology is observed in older patients. The probability of death from COVID-19 increases exponentially with age. In people older than 65 years, 8 of 10 COVID-19-associated deaths occur. Older patients with compromising diseases like heart failure, diabetes mellitus, hypertension, cancer, chronic obstructive pulmonary disease, and asthma have considerably high mortality rates. Governments and agencies worldwide have recognized the importance of protecting older adults during the COVID-19 pandemic.

Human-to-dog SARS-CoV-2 transmission: The mouth and nasal cavity samples of a 17-year-old Pomeranian dog were identified as “weakly positive” using qRT-PCR by the Agriculture Fisheries and Conservation Department of Hong Kong (AFCD) on 27 February 2020. Because the dog’s owner had been previously diagnosed with COVID-19, the dog was quarantined [113]. The dog showed no signs of the disease, and the rectal swab and fecal sample results remained negative. The RT-PCR was retested on 28 February, 2 March, 5 March, and 9 March 2020, and showed that there was a minute amount of SARS-CoV-2 RNA. In addition, there was a high correlation between these viral sequences because of the genomic data sequence for the dog and the close human contact on 12 March 2020. The antibody test was negative on 12 March 2020. The RT-PCR was negative on 12 and 13 March 2020, and the Pomeranian was released the next day. However, the dog died three days later, and unfortunately, the owner refused an autopsy test [114]. It was believed the death was not COVID-19 related, but instead, a result of several comorbidities. At the time of the study, there had not been support for owner-to-dog transmission, but researchers at the University of Hong Kong speculated that the dog possibly contracted COVID-19 from their owner. Moreover, in Hong Kong, a German shepherd tested positive for COVID-19 on 19 March 2020. This transmission was identified as human-to-dog transmission. The owner of the 2.5-year-old dog was also COVID-19 positive. Yet, during the quarantine period, the dog showed no respiratory clinical signs [54]. The AFCD announced that the dog’s nasal and oral samples were positive for RT-PCR. In another study, seventeen dogs and eight cats of owners suspected to be associated with COVID-19-positive cases were investigated. However, in this study, SARS-CoV-2 was positive in only two of the dogs [115]. It was believed that a dog with haemorrhagic diarrhea was exposed to the B.1.177 variant of SARS-CoV-2 that its human cohabitants had contracted in Spain. Unfortunately, the virus that infected its human host was not tested for sequencing [72].

The increased incidence of COVID-19 transmission from infected humans to animals, such as lions and tigers, is a growing concern, and the risk of transmission from pets to humans has been acknowledged and studied by public health officials. Cats with the virus but without significant clinical data serve as a silent COVID-19 vector to humans, as evidenced by several experimental reports [116]. It is speculated that the virus can be transmitted to humans through body fluids from pets because dogs are often near their owners, who hug, laugh, and play with them. Due to these findings, the CDC published guidelines to ensure the safety and health of pet owners. Children and older adults should not touch or be near animals, or the pet’s belongings such as beds, cages, animal waste, meals, and food supplies. In addition, hugging, cuddling, and holding animals should be discouraged.

In the event of a scratch or bite, urgent medical attention should be taken. Pets should also be monitored for health signs or unexpected changes in behavior. Because of uncertainty about the specific source of the virus, individuals who spend time in live animal product markets have been advised by the WHO to take universal precautions [117]. In addition, hands must be routinely washed with water and soap after contacting animals or their belongings, and the eyes, nose, and mouth must be protected when coming in contact with sick animals. Appropriate measures are required to prevent contact with soil or other surfaces that contain animal waste or fluids. All interactions with street animals, including dogs, cats, birds, and rodents should be avoided [118].

Growing detailed information on the risk of COVID-19 infection in animals, including pets and wildlife, is now available. However, when working with pets or other animals, sufficient measures like hand washing after contact with animals and animal waste should be taken [119]. In addition, pets must be thoroughly cleaned and maintained in good animal hygiene, otherwise, they may harbor germs that could make people sick. It is advised regular visits to the veterinarian or assistance from a veterinarian should be performed when animal health is a concern. Although only a few studies are available on the likelihood of SARS-CoV-2 transmission from humans to animals, confirmed and suspected individuals are advised to avoid contact with pets or other animals until the availability of further information on transmissibility is established [120]. It has also been recommended that an adequate hygienic environment be kept around the pet if no other family members care for the pet when the owner is infected. The infected person or owner must avoid or minimize contact with his or her. When handling and treating animals, good hygiene measures must be ensured, and special precautions must be taken when approaching the animal, for example, wearing a mask [96]. To maintain the health of animals during the pandemic, an emergency kit with sufficient food and medications should be prepared in advance [121]. According to the CDC, it is not necessary to exclude pets from households where COVID-19 is detected or suspected in one of the household members, but if the pets cannot be treated appropriately, the pets should be cared for by veterinarians. It should be noted that there is a distinction between animals contracting the virus and those transmitting it to humans. According to research experts, there is a low probability that people will contract COVID-19 from their pets. From person to person, the primary way of transmission includes exposure to saliva or other secretions from coughing, sneezing, and other body secretions.

The secondary route of transmission is via contaminated surfaces and objects. However, non-porous materials, such as doorknobs, can transmit the virus more than porous surfaces. Because animal skins fall into the porous surface category and are likely to entrap and uptake the virus, infection with the virus is difficult to achieve. However, it is possible that the virus can be transmitted from the body fluids of domestic animals to humans. As dogs are always around their owners, it is possible that they transmit the virus through kissing, licking, and playing. In addition, researchers have identified that the majority of infected ferrets and cats are asymptomatic meaning the obvious signs, such as sneezing, coughing, weight loss, or an increase in body temperature, would not be noted by the owners [122]; emphasizing the importance of owners taking caution when interacting with their pets as they may shed the virus without showing signs of having a COVID-19 infection. Given that SARS-CoV-2 has also been detected with human viral titers in animal feces, it is likely that the virus is transmitted to humans via infected feces and urine from pets. Any positive human cases of COVID-19 should refrain from contact with pets, as there are reports of COVID-19 transmission from infected humans to companion animals. But as previously stated, health authorities need to evaluate and investigate the likelihood of virus transmission from companion animals to humans. More research is needed to determine the various direct or indirect zoonotic transmission routes of SARS-CoV-2.

## 3. Human–Canine Interface: Human–Dog Behavior, Dog Adoptions, and Needed Care during Handling

### 3.1. Human–Canine Interface: Dog Adoption Boom Due to COVID-19 Pandemic

The pandemic of COVID-19 has sparked uncertainty and serious health and financial concerns. The ownership of companion animals, like dogs or cats, has previously been shown to have a positive impact on mental health. Animal interactions, particularly under stressful conditions, can counteract depression and anxiety. This interaction between humans and animals can strengthen peer-to-peer social relationships, as well as feelings of empathy, trust, and respect [123]. Even so, stress and poor well-being among dog owners have been shown to have negative effects on the well-being of their pets. Owing to the health, economic, and social burdens linked to COVID-19 and the reports of potential COVID-19 carriers, there could potentially be a dramatic increase in the virus being shed by dogs. To better understand the potential impact COVID-19 has had on human–dog relationships, researchers compiled and analyzed unique perspective and retrospective datasets on how people perceive and act in relation to dog adoption and abandonment during the COVID-19 pandemic social isolation, and the reciprocal relationship between dog owners and the welfare and health of their dogs [124,125]. Dog adoption rates increased significantly as social isolation worsened during the pandemic; the rate of abandonments did not change. It also became clear that a person’s worsening quality of life was associated with the perception that their dogs experienced a concurrent decline and that new behavioral problems were reported [124]. Given that humans and dogs are both social creatures, these findings have implications for the welfare of human–dog relationships during the COVID-19 epidemic. The One Welfare approach indicates a bidirectional link for the health and welfare of both humans and dogs [109,126,127,128]. As the climate continues to change, we will likely experience more disasters, including pandemics, emphasizing the need for more research on the human–animal relationships resulting from crises [129,130].

Furthermore, the COVID-19 shutdown also affected canines at shelters, likely due to changes in the shelter environment [95]. One of the biggest changes during this time was the use of foster care which dramatically increased from March to April 2020, but returned to normal levels by June 2020 [131]. The number of foster homes increased, but that impact was short-lived as home-based work declined. It was found that agencies could retain foster volunteers by delivering assistance that is human-centered rather than dog-centered, such as providing a more detailed history of the foster dogs or providing an experienced mentor to guide the new foster parent in the process of fostering [132]. Although working from home gave pet parents the opportunity to better attend to their pet’s needs, country-wide lockdowns impacted the human–dog relationship. The imposition of curfews and limited outdoor time substantially decreased the exercise time for dogs and their owners during the pandemic [133]. These increased times with owners early in the pandemic reduced separation-related behaviors (behaviors displayed by dogs when left alone at home). A study on the impact of separation behaviors found decreased signs from 22.1% in February 2020 to 17.2% in October 2020 when dogs were left alone. Nevertheless, there is some concern as new separation behaviors are being discovered in dogs, with almost 10% of dogs in the study displaying novel behaviors when left home alone [134].

### 3.2. Human–Canine Interface: Needed Care for Handling Dogs during the COVID-19 Pandemic

The risk of outside infection of canine owners and their dogs is much higher as many tend to go outside multiple times a day, with the risk of coming into contact with other dogs and people, sniffing and touching the ground in parks, or being in areas where another human or animal could have potentially infected the area. Pet owners who do take their dogs out are advised to maintain social distances, but even when maintained those in higher risk groups, such as older adults, need to keep precautions as popular areas to frequent with dogs, such as parks, can result in the transmission of SARS-CoV-2 [112].

When quarantined or under official lockdown due to COVID-19, owners significantly increase the time spent with their canine companion, resulting in more displays of affection such as petting, cuddling, and sharing a bed and food with them. From a medical standpoint, this typical type of affection towards one’s dogs is problematic as it provides ample opportunities for the virus to spread between humans and dogs, or vice versa. Because the virus is found in an infected animal’s saliva, an animal that cleans itself by licking, or an animal that licks a surface can potentially contaminate any surface it touches with SARS-CoV-2 [116].

Cautions around pets should still be taken, as studies have found people can be asymptomatic for COVID-19, and such findings are also noted in animals. In the absence of actual disease clinical signs, the possibility cannot be excluded for asymptomatic pets and livestock to excrete the virus as well as infect humans. However, according to Shi et al. [65], their viral load is much lower than in humans. The confirmed SARS-CoV-2 case in dogs in Hong Kong also did not show clinical signs. Even so, given the known zoonotic potential of corona-type viruses, caution should be exercised. For example, in a related virus, it is known that SARS-CoV-1 infects macaques and mice and in 2003 the SARS-CoV-1 was also known to cause zoonotic disease in humans as an animal virus. Similarly, the Middle East respiratory syndrome (MERS) initially emerged in Saudi Arabia in 2012 as a MERS CoV-induced respiratory disease. In fact, there are many diseases caused by coronaviruses that affect both companion animals and livestock. Diseases affecting companion animals include enteric coronaviruses, feline infectious peritonitis, and canine respiratory coronaviruses. Others affect livestock such as alpha coronaviruses, which cause mild respiratory or gastrointestinal illness, enteric coronaviruses in swine, coronaviruses in cattle, and infectious bronchitis viruses. With the current pandemic, the situation is changing rapidly, and given the current data, animals, including dogs, should be monitored for their potential in infecting humans with COVID-19.

To better monitor potential animal vectors, healthcare companies are developing laboratory tests to detect animals with SARS-CoV-2 infection. As the pandemic progresses and veterinarians test companion animals, our knowledge of the virus and its transmission from animal to animal or to humans is growing. As of 17 April 2020, two commercial laboratories in the United States reported testing RT-PCR on thousands of SARS-CoV-2 samples from dogs and cats, with no positive results [112,126]. PCR analyses of common respiratory pathogens in cats and dogs have been performed in South Korea, Canada, and Europe too [112,126], but minimal information is available on the proximity of these animals to human COVID-19-positive patients prior to testing. As SARS-CoV-2 is a pandemic according to the World Health Organization (WHO), the United States Department of Agriculture (USDA) reports all confirmed US animal infections to the World Organization for Animal Health (OIE). All current information is updated regularly and can be accessed by online sources from the AVMA [126], CDC [116], USDA [128], and OIE [135]. But more veterinary studies are necessary to identify different species and domestic animals that become infected with SARS-CoV-2 to determine if these animals will have clinical signs and/or an immune response. Additionally, it is crucial to determine if viral excretion in domestic species, such as dogs can be determined, from tears, urine, blood, saliva, or feces. Such studies will help identify what species could serve as virus reservoirs. Finally, extensive serosurveys of pets of verified COVID-19 patients provide further research opportunities in understanding the transmission of this disease between humans and animals.

Available research suggests that animals can either act as SARS-CoV-2 reservoirs or even potentially transmit COVID-19 to people in the household or people who are in close contact with animals. Therefore, pet owners are advised to follow certain safety guidelines to decrease the risk of contracting COVID-19 from their pet, or while caring for their pet. Standard hand-washing procedures should be followed before and after handling the animals. Due to the high mortality rate of older adults, they are advised to handle their pets in a more hygienic manner, similar to the “social distance” recommendation between people. According to CDC guidelines, older adults should not allow their pets to interact with other pets or other people outside the home. Indoor pets must be kept so that they do not communicate with other animals or people from outside the household. Dogs must be kept on a leash at a minimum distance of 2 m between humans and animals. Pet parks and public places should be avoided, as well as public transportation, where there are usually many people and dogs. For persons living alone during the COVID-19 lockdown, dogs are a safeguard against loneliness [136,137,138,139]. Even during the pandemic, most dog owners remained loyal to their dogs and put them first. This eases the dog owners` concerns and worries and improves communication between owners and veterinary teams [140]. In addition, dog walking improves the mental health of older adults, especially in stressful situations that can increase the risk of loneliness [138].

Besides the owners, veterinarians should also be careful. The potential causes of contracting COVID-19 in veterinarians include exposure to a SARS-CoV-2 infected dog, being a veterinarian working during lockdown hours, and not having disinfected the examination table following treatment of dogs in the clinic [141]. Additionally, the widespread of SARS-CoV-2 in the human population could lead to an increased risk of reverse zoonosis, in which the veterinarian could inadvertently infect a pet patient. Fortunately, the first canine COVID-19 subunit vaccine has been developed, including the recombinant SARS-CoV-2 protein of the entire S1 protein and RBD. Subunit vaccines only use the protein region, not the entire pathogen, and thus are considered a safer risk than whole-virus vaccines by eliminating the possibility of infection or replication through virus reverse mutation. These vaccines were subcutaneously injected twice at three-week intervals into several beagles, resulting in serum antibody titers comparable to those of vaccinated humans, which have been proven effective in protecting against SARS-CoV-2. Thus, domestic animal vaccination, like dog vaccination, could preclude the reverse zoonosis scenario, protecting pets, owners, and animal healthcare workers from SARS-CoV-2 [142].

### 3.3. Human–Canine Interface: Cardiac Failure Is a Possible Cause of Death for Dogs That Requires in-Depth Investigation, as Learned from COVID-19 in Humans

As noted, one of the dogs (a 17-year-old Pomeranian puppy) died shortly after being diagnosed with COVID-19, without showing respiratory signs, with its death being believed to be unrelated to its COVID-19 infection [128]. It is worth noting, though, that one in five COVID-19 patients has cardiac damage resulting in cardiac failure and death, including patients without respiratory distress syndrome; cardiac arrest is the most prevalent cause of mortality in COVID-19 patients. Along with its hypoxic damage, SARS-CoV-2 has a high propensity to infect the myocardium. As a cellular entry receptor into cardiac myocytes and coronary arteries, SARS-CoV-2 uses ACE-2 extensively. Even though it was deemed that the dog infected with COVID-19 did not die from it, it did die from heart failure [143]. Genetically impaired cardiac resilience may pose an increased risk of COVID-19 deaths in infected dogs. Therefore, the recommendation is that SARS-CoV-2 may require cardiac pathology that needs to be recognized by veterinarians and that they should more closely evaluate the cardiac signs even if the COVID-19-positive dog does not show clinical respiratory signs. To classify SARS-CoV-2 as a cause of cardiac failure in COVID-19-infected domestic dogs, thorough pathological examinations must be carried out. Recently, systolic dysfunction has been shown to aggravate myocardial damage in a COVID-19-positive dog [144].

Historically, dogs are used as important animal models for studying heart failure and chronic heart disease in veterinary and cardiovascular research [145]. Many universities and pharmaceutical sector researchers who regularly handle dogs may be vulnerable to COVID-19. The likelihood of contacting the body fluids of potential virus-containing animals is increased in the laboratory [146], particularly during procedures involving invasive surgery. Researchers should undergo appropriate safety training to avoid SARS-CoV-2 transmission and carefully follow the laboratory standards for handling hazardous waste and biological specimens. In addition to the cardiac lesions, a post-acute SARS-CoV-2 sequela of infection in a dog with a breed predisposed to Canine Idiopathic Pulmonary Fibrosis was hypothesized and supported by comparing the identified lesions with those described in humans [147].

## 4. Human–Canine Interface: Possible Roles of Dogs in Controlling COVID-19 Pandemic

### 4.1. Detection Dogs as a Rapid and Reliable Diagnostic Approach for COVID-19 Patients

The current COVID-19 pandemic emphasizes the need for rapid and accurate testing of asymptomatic and symptomatic carriers to effectively contain the spread of infection [148]. Current diagnostic methods commonly use nasal and nasopharyngeal swabs to detect the pathogen, which requires a qualified person to perform RT-PCR. In addition, RT-PCR is time-consuming and expensive, especially in developing countries; therefore, it is usually targeted at patients with specific COVID-19 clinical signs [148]. Alternatively, it has been found that dogs can be trained to diagnose diseases through scent. They can detect odors 10,000–100,000 times better than humans. Several investigations have demonstrated their exceptional olfactory ability to identify individuals with infectious or other non-infectious diseases such as various metabolic diseases (e.g., hypoglycemia and diabetes), neoplasms (e.g., colon, bladder, and melanoma) [149,150,151], viral infections, bacterial diseases, and malaria [152,153,154,155]. Pathogenic odors can be detected by certain patterns of volatile organic compounds (VOCs) that represent odor casts. Unlike bacteria, viruses do not have their own metabolism, and the release of VOCs occurs because of host metabolic processes by infected cells [156]. Several technical methods have effectively leveraged the identification of VOCs to distinguish infectious diseases, however, they have not been used routinely in clinical practice [157]. In recent research, dogs have been used to reliably separate samples from COVID-19 patients from healthy controls in real-time, as they were rapidly trained on tracheobronchial secretions or saliva from COVID-19 patients [158]. In addition, several studies have used trained sniffer dogs to distinguish between the sweat of COVID-19-positive and negative individuals [159,160,161,162,163]. The protocols used for training dogs included olfactory conditioning and discrimination research [160]. According to recent findings from the United Arab Emirates, trained dogs can diagnose COVID-19 with high accuracy (92% accuracy) [149,150]. They could diagnose COVID-19 infections in asymptomatic individuals with higher sensitivity than RT-PCR, do not require high technical effort (less invasive), and are faster, cheaper, more flexible, efficient, reliable, and easier to handle [157,164].

Another advantage to using dogs in detecting COVID-19 is that they can be used in various settings involving public facilities such as airports, borders, sporting events, and other large crowds as an alternative or complement to the standard RT-PCR [164]. In addition, they can be used as a mass detection tool in countries with limited access to diagnostic testing to identify infected individuals. Further research is required for a better understanding of the capabilities of sniffer dogs to detect viral respiratory diseases and to highlight possible limitations of using dogs. In a recent study, researchers found dogs could rapidly and correctly detect confirmed COVID-19-positive and -negative people, 86% and 92.9%, respectively, which is comparable to results found with RT-PCR [149]. All dogs in the testing phase identified SARS-CoV-2 with high accuracy; the diagnostic overall sensitivity was 98%, and the specificity was 92% for 584 individuals (76% negative samples and 24% positive samples). In the follow-up phase, one dog was able to screen 153 COVID-19 patients at one hospital with 96% diagnostic sensitivity and 100% specificity [165]. Further performance of trained dogs was investigated to identify an individual’s status of SARS-CoV-2 infection using axillary sweat-stained wipes and exhaled air recovered in surgical masks of individuals with mild to severe COVID-19 infections, asymptomatic individuals, and vaccinated individuals. Newly trained dogs examined 886 sweat samples from 241 individuals and on average they were able to detect SARS-CoV-2 at an 89.6% diagnostic sensitivity and a specificity of 83.9%, even when low viral load individuals were involved in the analysis. It is worth noting that some of the dogs were better at detecting the virus compared to others, with a range from 58–80% sensitivity and 64–88% specificity. The authors speculate this variation is in part due to the short 4.5-month training period and that sweat was only collected for 3 min from participating study patients [166]. Even so when 207 sweat samples from vaccinated individuals were considered, the dogs’ specificity and sensitivity were 86.0% and 85.7%, respectively, although the probability of false positives was higher during the two weeks directly after COVID-19 vaccination. The performance of dogs shows promise, as their detection capacity is anticipated to improve upon prolonged exposure of the gauze sample in the saliva and sweat of COVID-19 patients. Achieving the sensitivity and specificity range required by WHO for an antigen test was the main objective, but only one dog achieved the needed ≥80% sensitivity and none were near the ≥97% specificity [166]; still, such results are promising and suggesting improved training of dogs might meet the desired criterion. In another study, the persistence of SARS-CoV-2 in patients with COVID-19 was investigated using detection dogs and scents from axillary sweat samples [167]. Using a randomized and double-blind methodology, seven search and rescue dogs tested 218 axillary sweat samples (62 positives and 156 negatives). Sensitivity levels ranged between 87% and 94%, and specificity values ranged between 78% and 92%, including four dogs that scored greater than 90% [168]. Those trained sniffer dogs screened passengers’ samples with a high level of accuracy in a large, randomized, controlled, triple-blind validation study at an international airport [169]. Canine olfaction as a potential alternative to RT-PCR in diagnosing SARS-CoV-2 infections seems to have great promise [170]. Additionally, studies have found that any breed or mixed breed of dogs can be trained to perform COVID-19 screening [159,171,172,173,174]. The breeds such as German shepherds, Belgian Malinois, Dutch shepherds, pit bull dogs, golden retrievers, and Nordic sled dogs have been previously used [172]. Furthermore, dogs used in these studies were previously trained for search and rescue, explosive detection, security, epilepsy, and colon cancer detection dogs [159,164,168,173].

### 4.2. Tested Anti-Inflammatory Drugs for Canine Coronavirus Can Be Helpful for COVID-19 in Humans

Vigorous immune responses, such as cytokine storms, are linked to severe COVID-19 cases and deaths [175]. Thimins process involves exposure to similar antigenic epitopes and is also influenced by coronaviruses associated with taxonomies from some animal species such as pigs, dogs, and cattle [176,177]. Two likely anti-inflammatory agents, resveratrol and indomethacin, are potent antioxidants that have been shown to suppress canine coronavirus (CCoV) levels in dogs and exert antiviral effects on many other viruses [178]. Indomethacin inhibits COX-1 and COX-2 to treat various inflammatory conditions. It is not expensive nor selective for cyclooxygenase (COX). It was shown that indomethacin exhibits antiviral action against CCoV in Vero cells in vitro and at a low dosage (1 mg/kg) in vivo in CCoV-infected dogs [153]. The authors noted that indomethacin was a more potent anti-inflammatory drug than aspirin. They suggested the use of resveratrol and indomethacin as possible therapeutic adjuncts for COVID-19. Additional anti-inflammatory drugs may be critical in treating COVID-19 patients because they improve clinical outcomes by controlling the severe inflammation resulting from the cytokine storms that hurt the circulatory, renal, respiratory, immune, and other systems. In addition to the anti-inflammatory effect, some have antiviral effects on viral replication. In addition to these two drugs, the in vitro absorption, distribution, metabolism, and excretion (ADME) properties and preclinical pharmacokinetics (PK) of GS-441524 and Emvododstat had been tested as potential agents in a range of species, including dogs [154,155].

## 5. Conclusions

Globally, dogs are one of the most popular pets and many dog owners admit to sharing food, cuddling, sharing their beds, and other forms of affection that bring them in close contact with their dogs on a regular basis. The COVID-19 pandemic only increased dog ownership and affinity with many animal shelters becoming empty as dogs were adopted or fostered out, although this trend did not last the whole pandemic. The rise of dog ownership and the increased time owners are spending with their canine companions are of concern given the zoonotic history of coronaviruses, and the known zoonotic transmissibility of COVID-19.

In this review, we highlight that dogs can become infected with COVID-19, but research is needed to determine if dogs can spread the SARS-CoV-2 virus to humans. One major challenge to determining if dogs are vectors is that they are asymptomatic and thus molecular, or serology tests are needed to confirm infection. Rapid diagnostic tests for dogs and close monitoring of dogs that have been exposed to COVID-19-positive people are needed to improve our understanding of their ability to transmit the SARS-CoV-2 virus to humans. Although there is a concern about dogs being potential vectors in spreading SARS-CoV-2, other studies have highlighted the benefits of dogs during the COVID-19 pandemic. Dogs, like other pets, are known to reduce stress and anxiety for their owners. Sniffer dogs can be used as a rapid and reliable approach for diagnosing SARS-CoV-2 in large populations, which is essential for controlling and interrupting COVID-19 chains of infection, although they are not as accurate as antigen tests. Future studies should continue to examine the human–canine interface and how pandemics affect this relationship. Such research will shed light on the benefits and possible consequences of living in close quarters with our canine companions.

## Figures and Tables

**Table 1:** Studies of COVID-19 in dogs and the country at which the study was performed.

Study of COVID-19 in Dogs	Country	Number of Animals and Positive Animals	Method of Detection	Infections Type	Reference
Two dogs (no clinical sign, detected SARS-CoV-2 RNA and neutralizing antibodies)	China	15 (2)	Real-time RT-PCR, sequencing, and viral isolation	Natural	[54]
Human-to-dog transmission. Viral RNA shedding could be observed.	France	One infected dog	RT_qPCR PCR ELISA automated Western blotting (WB) assays WGS	Natural	[56]
A longitudinal study in Texas	USA	59 (one dog was verified by PCR and sequencing) seven dogs had neutralizing antibodies	RT_qPCR virus isolation sequencing virus neutralization test (VNT)	Natural	[99]
Infection of SARS-CoV-2 in a dog resulting in haemorrhagic diarrhea	Spain	One dog	RT-PCR sequencing	Natural	[72]
High prevalence of antibodies in dogs from households with SARS-CoV-2 +ve patients	France	13 (5 were seropositive)	FACS multiplex microsphere immunoassay	Natural	[98]
Survey to detect SARS-CoV-2 among pets of owners positive to SARS-CoV-2 across 15 communities in Wuhan.	China	9 dogs (One dog tested as positive showing high ELISA-OD values for IgG)	RT_qPCR ELISA Plaque reduction neutralization test (PRNT)	Natural	[96]
In a large-scale study in Italy, no dogs tested PCR-positive. However, 3.3% (15/451) of dogs had seroconversion for SARS-CoV-2, with dogs housed with SARS-CoV-2 infected patients being significantly to be positive than the COVID-19 negative households.	Italy	451 dogs (neutralizing antibodies were identified in 15 dogs (3.3%)	RT_qPCR PRNT	Natural	[100]
A survey of 487 dogs showed serological negative to SARS-CoV-2.	China	487 (Serologically tested to be negative)	double-antigen sandwich ELISA	Natural	[105]
Stray dogs seropositivity in Brazil	Brazil	47 dogs (serum samples of a stray cat and a stray dog presented neutralizing antibodies)	PRNT RT_qPCR	Natural	[68]
SARS-CoV-2 infection within dogs in Thailand	Thailand	three out of 35 dogs	indirect ELISA and VNT RT_qPCR WGS	Natural	[106]
SARS-CoV-2 infection and seropositivity in Utah and Wisconsin	Utah and Wisconsin (USA)	37 dogs (one dog RT_qPCR, one dog’s fur swabs) 48 (12% are seropositive (4 dogs)	RT_qPCR, sequencing VNT	Natural	[107]
SARS-CoV-2 infection within dogs in Croatia	Croatia	1038 serum samples from dogs (Out of 149 ELISA positive samples, 23 had neutralizing antibody) 87 dogs from household (43.9%) were ELISA positive, and neutralizing antibody was detected in 25.64% of 87 dogs from household dogs)	microneutralization test ELISA	Natural	[108]
SARS-CoV-2 infection within dogs in France	France	165 (neutralizing antibodies are detected in 5.4% of the dogs (9/165)	ELISA HAT VNT	Natural	[109]
No viral RNA was diagnosed in 12 dogs residing in northern Spain with individuals reported to be infected	Northern Spain	12 dogs (Viral RNA was not observed)	RT_qPCR	Natural	[60]
In France, an investigation conducted at a veterinary campus has revealed that cats and dogs that were closely in contact with patients with COVID-19 did not contract SARS-CoV-2.	France	12 during the epidemic and 55 dogs before the epidemic (0)	Luciferase Immuno-Precipitation System (LIPSPCR)	Natural	[57]
Experimentally infected five SARS-CoV-2 beagle dogs	China	5 experimentally infected and uninoculated two dogs Two of the four inoculated dogs developed antibodies; however, neither the remaining two inoculated dogs nor the other two contact-exposed dogs produced anti-SARS-CoV-2 antibodies.	Rectal swabs from two virus-inoculated dogs were positive for viral RNA at 2 dpi. No clinical signs, no replication or transmission to in-contact animals and no pathological necropsy changes but only antibody response is detected.	Experimental	[65]

## Data Availability

Not applicable.

## References

[1] El-Sayed A., Aleya L., Kamel M. (2021). COVID-19: A new emerging respiratory disease from the neurological perspective. Environ. Sci. Pollut. Res..

[2] El-Sayed A., Abdel-Daim M.M., Kamel M. (2021). Causes of respiratory failure in COVID-19 patients. Environ. Sci. Pollut. Res..

[3] Aboubakr H.A., Sharafeldin T.A., Goyal S.M. (2021). Stability of SARS-CoV-2 and other coronaviruses in the environment and on common touch surfaces and the influence of climatic conditions: A review. Transbound. Emerg. Dis..

[4] Liu Y., Gayle A.A., Wilder-Smith A., Rocklöv J. (2020). The reproductive number of COVID-19 is higher compared to SARS coronavirus. J. Travel Med..

[5] Tazerji S.S., Duarte P.M., Rahimi P., Shahabinejad F., Dhakal S., Malik Y.S., Shehata A.A., Lama J., Klein J., Safdar M. (2020). Transmission of severe acute respiratory syndrome coronavirus 2 (SARS-CoV-2) to animals: An updated review. J. Transl. Med..

[6] Swelum A.A., Shafi M.E., Albaqami N.M., El-Saadony M.T., Elsify A., Abdo M., Taha A.E., Abdel-Moneim A.-M.E., Al-Gabri N.A., Almaiman A.A. (2020). COVID-19 in Human, Animal, and Environment: A Review. Front. Vet. Sci..

[7] Mahdy M.A.A., Younis W., Ewaida Z. (2020). An Overview of SARS-CoV-2 and Animal Infection. Front. Vet. Sci..

[8] Defo Deeh P.B., Kayri V., Orhan C., Sahin K. (2020). Status of Novel Coronavirus Disease 2019 (COVID-19) and Animal Production. Front. Vet. Sci..

[9] Alluwaimi A.M., Alshubaith I.H., Al-Ali A.M., Abohelaika S. (2020). The Coronaviruses of Animals and Birds: Their Zoonosis, Vaccines, and Models for SARS-CoV and SARS-CoV2. Front. Vet. Sci..

[10] Irian M. (2020). COVID-19, Your Pet and Other Animals: Are You at Risk?. MEDICC Rev..

[11] Haider N., Rothman-Ostrow P., Osman A.Y., Arruda L.B., Macfarlane-Berry L., Elton L., Thomason M.J., Yeboah-Manu D., Ansumana R., Kapata N. (2020). COVID-19—Zoonosis or Emerging Infectious Disease?. Front. Public Health.

[12] Hashem N.M., González-Bulnes A., Rodriguez-Morales A.J. (2020). Animal Welfare and Livestock Supply Chain Sustainability Under the COVID-19 Outbreak: An Overview. Front. Vet. Sci..

[13] Anisuzzaman, Haque Z.F., Hossain M.T. (2022). COVID-19 Pandemic: Animal Cross Talk and Comparison between nSARS-CoV-2 and Animal Coronaviruses.

[14] Chandler J.C., Bevins S.N., Ellis J.W., Linder T.J., Tell R.M., Jenkins-Moore M., Root J.J., Lenoch J.B., Robbe-Austerman S., DeLiberto T.J. (2021). SARS-CoV-2 exposure in wild white-tailed deer (*Odocoileus virginianus*). Proc. Natl. Acad. Sci. USA.

[15] Stout A.E., André N.M., Jaimes J.A., Millet J.K., Whittaker G.R. (2020). Coronaviruses in cats and other companion animals: Where does SARS-CoV-2/COVID-19 fit?. Vet. Microbiol..

[16] Cui S., Liu Y., Zhao J., Peng X., Lu G., Shi W., Pan Y., Zhang D., Yang P., Wang Q. (2022). An Updated Review on SARS-CoV-2 Infection in Animals. Viruses.

[17] Sharun K., Dhama K., Pawde A.M., Gortázar C., Tiwari R., Bonilla-Aldana D.K., Rodriguez-Morales A.J., de La Fuente J., Michalak I., Attia Y.A. (2021). SARS-CoV-2 in animals: Potential for unknown reservoir hosts and public health implications. Vet. Q..

[18] Meekins D.A., Gaudreault N.N., Richt J.A. (2021). Natural and Experimental SARS-CoV-2 Infection in Domestic and Wild Animals. Viruses.

[19] Michelitsch A., Wernike K., Ulrich L., Mettenleiter T.C., Beer M. (2021). SARS-CoV-2 in animals: From potential hosts to animal models. Adv. Virus Res..

[20] Nerpel A., Yang L., Sorger J., Käsbohrer A., Walzer C., Desvars-Larrive A. (2022). SARS-ANI: A global open access dataset of reported SARS-CoV-2 events in animals. Sci. Data.

[21] SARS-ANI VIS. https://vis.csh.ac.at/sars-ani/#outcomes.

[22] Lam T.T.-Y., Jia N., Zhang Y.-W., Shum M.H.-H., Jiang J.-F., Zhu H.-C., Tong Y.-G., Shi Y.-X., Ni X.-B., Liao Y.-S. (2020). Identifying SARS-CoV-2-related coronaviruses in Malayan pangolins. Nature.

[23] Ye Z.-W., Yuan S., Yuen K.-S., Fung S.-Y., Chan C.-P., Jin D.-Y. (2020). Identifying SARS-CoV-2-related coronaviruses in Malayan pangolins. Int. J. Biol. Sci..

[24] Bengis R.G., Leighton F.A., Fischer J.R., Artois M., Morner T., Tate C.M. (2004). The role of wildlife in emerging and re-emerging zoonoses. Rev. Sci. Tech. L’oie.

[25] Woolhouse M.E.J. (2002). Population biology of emerging and re-emerging pathogens. Trends Microbiol..

[26] El-Sayed A., Abdel-Daim M.M., Kamel M. (2021). Zoonotic and anthropozoonotic potential of COVID-19 and its implications for public health. Environ. Sci. Pollut. Res..

[27] El-Sayed A., Kamel M. (2021). Coronaviruses in humans and animals: The role of bats in viral evolution. Environ. Sci. Pollut. Res..

[28] Durr P., Gatrell A. (2004). GIS and Spatial Analysis in Veterinary Science.

[29] Carella E., Orusa T., Viani A., Meloni D., Borgogno-Mondino E., Orusa R. (2022). An Integrated, Tentative Remote-Sensing Approach Based on NDVI Entropy to Model Canine Distemper Virus in Wildlife and to Prompt Science-Based Management Policies. Animals.

[30] Norstrøm M. (2001). Geographical Information System (GIS) as a tool in surveillance and monitoring of animal diseases. Acta Vet. Scand..

[31] de Marinis P., de Petris S., Sarvia F., Manfron G., Momo E.J., Orusa T., Corvino G., Sali G., Borgogno E.M. (2021). Supporting Pro-Poor Reforms of Agricultural Systems in Eastern DRC (Africa) with Remotely Sensed Data: A Possible Contribution of Spatial Entropy to Interpret Land Management Practices. Land.

[32] Pfeiffer D.U. (2004). Geographical information science and spatial analysis in animal health. GIS and Spatial Analysis in Veterinary Science.

[33] Orusa T., Mondino E.B. (2019). Landsat 8 thermal data to support urban management and planning in the climate change era: A case study in Torino area, NW Italy. Remote Sensing Technologies and Applications in Urban Environments IV.

[34] Orusa T., Orusa R., Viani A., Carella E., Borgogno Mondino E. (2020). Geomatics and EO Data to Support Wildlife Diseases Assessment at Landscape Level: A Pilot Experience to Map Infectious Keratoconjunctivitis in Chamois and Phenological Trends in Aosta Valley (NW Italy). Remote Sens..

[35] Orusa T., Borgogno Mondino E. (2021). Exploring Short-Term Climate Change Effects on Rangelands and Broad-Leaved Forests by Free Satellite Data in Aosta Valley (Northwest Italy). Climate.

[36] Kuldeep D., Verma A.K., Ruchi T., Sandip C., Kranti V., Sanjay K., Rajib D., Karthik K., Rajendra S., Muhammad M. (2013). A perspective on applications of Geographical Information System (GIS): An advanced tracking tool for disease surveillance and monitoring in veterinary epidemiology. Adv. Anim. Vet. Sci..

[37] Fayisa W.O. (2020). Review on The Importance of Geographic Information System (Gis) In Epidemiology: In Prevention and Control of Animal Disease. Int. J. Vet. Sci. Res..

[38] Rinaldi L., Musella V., Biggeri A., Cringoli G. (2006). New insights into the application of geographical information systems and remote sensing in veterinary parasitology. Geospat. Health.

[39] Garg N., Ahmad F.J., Kar S. (2022). Recent advances in loop-mediated isothermal amplification (LAMP) for rapid and efficient detection of pathogens. Curr. Res. Microb. Sci..

[40] Wang J., Ranjbaran M., Ault A., Verma M.S. (2023). A loop-mediated isothermal amplification assay to detect Bacteroidales and assess risk of fecal contamination. Food Microbiol..

[41] Wang J., Davidson J.L., Kaur S., Dextre A.A., Ranjbaran M., Kamel M.S., Athalye S.M., Verma M.S. (2022). Paper-Based Biosensors for the Detection of Nucleic Acids from Pathogens. Biosensors.

[42] Pascual-Garrigos A., Maruthamuthu M.K., Ault A., Davidson J.L., Rudakov G., Pillai D., Koziol J., Schoonmaker J.P., Johnson T., Verma M.S. (2021). On-farm colorimetric detection of *Pasteurella multocida*, Mannheimia haemolytica, and Histophilus somni in crude bovine nasal samples. Vet. Res..

[43] Davidson J.L., Wang J., Maruthamuthu M.K., Dextre A., Pascual-Garrigos A., Mohan S., Putikam S.V.S., Osman F.O.I., McChesney D., Seville J. (2021). A paper-based colorimetric molecular test for SARS-CoV-2 in saliva. Biosens. Bioelectron. X.

[44] Mohan S., Pascual-Garrigos A., Brouwer H., Pillai D., Koziol J., Ault A., Schoonmaker J., Johnson T., Verma M.S. (2021). Loop-Mediated Isothermal Amplification for the Detection of *Pasteurella multocida*, *Mannheimia haemolytica*, and *Histophilus somni* in Bovine Nasal Samples. ACS Agric. Sci. Technol..

[45] Wang J., Dextre A., Pascual-Garrigos A., Davidson J.L., McChesney D., Seville J., Verma M.S. (2021). Fabrication of a paper-based colorimetric molecular test for SARS-CoV-2. MethodsX.

[46] Ranjbaran M., Verma M.S. (2022). Microfluidics at the interface of bacteria and fresh produce. Trends Food Sci. Technol..

[47] Avendaño C., Patarroyo M.A. (2020). Loop-Mediated Isothermal Amplification as Point-of-Care Diagnosis for Neglected Parasitic Infections. Int. J. Mol. Sci..

[48] Nadeem M.S., Zamzami M.A., Choudhry H., Murtaza B.N., Kazmi I., Ahmad H., Shakoori A.R. (2020). Origin, Potential Therapeutic Targets and Treatment for Coronavirus Disease (COVID-19). Pathogens.

[49] Dhama K., Khan S., Tiwari R., Sircar S., Bhat S., Malik Y.S., Singh K.P., Chaicumpa W., Bonilla-Aldana D.K., Rodriguez-Morales A.J. (2020). Coronavirus Disease 2019–COVID-19. Clin. Microbiol. Rev..

[50] Satarker S., Nampoothiri M. (2020). Structural Proteins in Severe Acute Respiratory Syndrome Coronavirus-2. Arch. Med. Res..

[51] Du L., He Y., Zhou Y., Liu S., Zheng B.-J., Jiang S. (2009). The spike protein of SARS-CoV—A target for vaccine and therapeutic development. Nat. Rev. Microbiol..

[52] Dhama K., Patel S.K., Sharun K., Pathak M., Tiwari R., Yatoo M.I., Malik Y.S., Sah R., Rabaan A.A., Panwar P.K. (2020). SARS-CoV-2 jumping the species barrier: Zoonotic lessons from SARS, MERS and recent advances to combat this pandemic virus. Travel Med. Infect. Dis..

[53] Hobbs E.C., Reid T.J. (2020). Animals and SARS-CoV-2: Species susceptibility and viral transmission in experimental and natural conditions, and the potential implications for community transmission. Transbound. Emerg. Dis..

[54] Sit T.H.C., Brackman C.J., Ip S.M., Tam K.W.S., Law P.Y.T., To E.M.W., Yu V.Y.T., Sims L.D., Tsang D.N.C., Chu D.K.W. (2020). Infection of dogs with SARS-CoV-2. Nature.

[55] Abdel-Moneim A.S., Abdelwhab E.M. (2020). Evidence for SARS-CoV-2 Infection of Animal Hosts. Pathogens.

[56] Medkour H., Catheland S., Boucraut-Baralon C., Laidoudi Y., Sereme Y., Pingret J.-L., Million M., Houhamdi L., Levasseur A., Cabassu J. (2021). First evidence of human-to-dog transmission of SARS-CoV-2 B.1.160 variant in France. Transbound. Emerg. Dis..

[57] Temmam S., Barbarino A., Maso D., Behillil S., Enouf V., Huon C., Jaraud A., Chevallier L., Backovic M., Pérot P. (2020). Absence of SARS-CoV-2 infection in cats and dogs in close contact with a cluster of COVID-19 patients in a veterinary campus. One Health.

[58] Hernández M., Abad D., Eiros J.M., Rodríguez-Lázaro D. (2020). Are Animals a Neglected Transmission Route of SARS-CoV-2?. Pathogens.

[59] WHO. https://www.who.int/docs/default-source/coronaviruse/real-time-rt-pcr-assays-for-the-detection-of-sars-cov-2-institut-pasteur-paris.pdf?sfvrsn=3662fcb6_2.

[60] Ruiz-Arrondo I., Portillo A., Palomar A.M., Santibáñez S., Santibáñez P., Cervera C., Oteo J.A. (2021). Detection of SARS-CoV-2 in pets living with COVID-19 owners diagnosed during the COVID-19 lockdown in Spain: A case of an asymptomatic cat with SARS-CoV-2 in Europe. Transbound Emerg Dis..

[61] Carpenter A., Ghai R., Gary J., Ritter J., Carvallo F., Diel D.D., Martins M., Murphy J., Schroeder B., Brightbill K. (2021). Determining the Role of Natural SARS-CoV-2 Infection in the Death of Ten Domestic Pets.

[62] Rutherford C., Kafle P., Soos C., Epp T., Bradford L., Jenkins E. (2022). Investigating SARS-CoV-2 Susceptibility in Animal Species: A Scoping Review. Environ. Health Insights.

[63] Meisner J., Baszler T.V., Kuehl K.H., Ramirez V., Baines A., Frisbie L.A., Lofgren E.T., De Avila D.M., Wolking R.M., Bradway D.S. (2021). Household Transmission of SARS-CoV-2 from Humans to Dogs in Washington and Idaho: Burden and Risk Factors. bioRxiv.

[64] Lyoo K.-S., Yeo Y., Lee S.-G., Yeom M., Bae E.-H., Lee J.-Y., Kim K.-C., Song D. (2021). Pathogenicity of SARS-CoV-2 and MERS-CoV in Beagle Dogs.

[65] Shi J., Wen Z., Zhong G., Yang H., Wang C., Huang B., Liu R., He X., Shuai L., Sun Z. (2020). Susceptibility of ferrets, cats, dogs, and other domesticated animals to SARS–coronavirus 2. Science.

[66] OIE, World Organisation for Animal Health SARS-COV-2 in Animals—Situation Report 2 2021. https://www.oie.int/app/uploads/2021/07/sars-cov-2-situation-report-2.pdf.

[67] Jairak W., Charoenkul K., Chamsai E., Udom K., Chaiyawong S., Hangsawek A., Waenkaew S., Mungaomklang A., Tangwangvivat R., Amonsin A. (2022). Survey of SARS-CoV-2 in dogs and cats in high-risk areas during the second wave of COVID-19 outbreak, Thailand. Zoonoses Public Health.

[68] Dias H.G., Resck M.E.B., Caldas G.C., Resck A.F., da Silva N.V., Dos Santos A.M.V., Sousa T.d.C., Ogrzewalska M.H., Siqueira M.M., Pauvolid-Corrêa A. (2021). Neutralizing antibodies for SARS-CoV-2 in stray animals from Rio de Janeiro, Brazil. PLoS ONE.

[69] Chetboul V., Foulex P., Kartout K., Klein A.M., Sailleau C., Dumarest M., Delaplace M., Gouilh M.A., Mortier J., Le Poder S. (2021). Myocarditis and Subclinical-Like Infection Associated With SARS-CoV-2 in Two Cats Living in the Same Household in France: A Case Report With Literature Review. Front. Vet. Sci..

[70] Ferasin L., Fritz M., Ferasin H., Becquart P., Corbet S., Ar Gouilh M., Legros V., Leroy E.M. (2021). Infection with SARS-CoV-2 variant B.1.1.7 detected in a group of dogs and cats with suspected myocarditis. Vet. Rec..

[71] Barroso-Arévalo S., Rivera B., Domínguez L., Sánchez-Vizcaíno J.M. (2021). First Detection of SARS-CoV-2 B.1.1.7 Variant of Concern in an Asymptomatic Dog in Spain. Viruses.

[72] Padilla-Blanco M., Vega S., Enjuanes L., Morey A., Lorenzo T., Marín C., Ivorra C., Maiques E., Rubio V., Rubio-Guerri C. (2022). Detection of SARS-CoV-2 in a dog with hemorrhagic diarrhea. BMC Vet. Res..

[73] Sun S.-H., Chen Q., Gu H.-J., Yang G., Wang Y.-X., Huang X.-Y., Liu S.-S., Zhang N.-N., Li X.-F., Xiong R. (2020). A Mouse Model of SARS-CoV-2 Infection and Pathogenesis. Cell Host Microbe.

[74] Hoffmann M., Kleine-Weber H., Schroeder S., Krüger N., Herrler T., Erichsen S., Schiergens T.S., Herrler G., Wu N.-H., Nitsche A. (2020). SARS-CoV-2 Cell Entry Depends on ACE2 and TMPRSS2 and Is Blocked by a Clinically Proven Protease Inhibitor. Cell.

[75] Lai C.-C., Shih T.-P., Ko W.-C., Tang H.-J., Hsueh P.-R. (2020). Severe acute respiratory syndrome coronavirus 2 (SARS-CoV-2) and coronavirus disease-2019 (COVID-19): The epidemic and the challenges. Int. J. Antimicrob. Agents.

[76] Chiocchetti R., Galiazzo G., Fracassi F., Giancola F., Pietra M. (2020). ACE2 Expression in the Cat and the Tiger Gastrointestinal Tracts. Front. Vet. Sci..

[77] Tiwari R., Dhama K., Sharun K., Iqbal Yatoo M., Malik Y.S., Singh R., Michalak I., Sah R., Bonilla-Aldana D.K., Rodriguez-Morales A.J. (2020). COVID-19: Animals, veterinary and zoonotic links. Vet. Q..

[78] Luan J., Lu Y., Jin X., Zhang L. (2020). Spike protein recognition of mammalian ACE2 predicts the host range and an optimized ACE2 for SARS-CoV-2 infection. Biochem. Biophys. Res. Commun..

[79] Conceicao C., Thakur N., Human S., Kelly J.T., Logan L., Bialy D., Bhat S., Stevenson-Leggett P., Zagrajek A.K., Hollinghurst P. (2022). SARS-CoV-2 Spike has broad tropism for mammalian ACE2 proteins yet exhibits a distinct pattern of receptor usage when compared to other β-coronavirus Spike proteins. Access Microbiol..

[80] Mathavarajah S., Dellaire G. (2020). Lions, Tigers and Kittens too: ACE2 and susceptibility to CoVID-19. Evol. Med. Public Health.

[81] Demogines A., Farzan M., Sawyer S.L. (2012). Evidence for ACE2-Utilizing Coronaviruses (CoVs) Related to Severe Acute Respiratory Syndrome CoV in Bats. J. Virol..

[82] Melin A.D., Janiak M.C., Marrone F., Arora P.S., Higham J.P. (2020). Comparative ACE2 variation and primate COVID-19 risk. bioRxiv.

[83] Qiu Y., Zhao Y.-B., Wang Q., Li J.-Y., Zhou Z.-J., Liao C.-H., Ge X.-Y. (2020). Predicting the angiotensin converting enzyme 2 (ACE2) utilizing capability as the receptor of SARS-CoV-2. Microbes Infect..

[84] Sun J., He W.-T., Wang L., Lai A., Ji X., Zhai X., Li G., Suchard M.A., Tian J., Zhou J. (2020). COVID-19: Epidemiology, evolution, and cross-disciplinary perspectives. Trends Mol. Med..

[85] Sang E.R., Tian Y., Gong Y., Miller L.C., Sang Y. (2020). Integrate structural analysis, isoform diversity, and interferon-inductive propensity of ACE2 to predict SARS-CoV2 susceptibility in vertebrates. Heliyon.

[86] Brooke G.N., Prischi F. (2020). Structural and functional modelling of SARS-CoV-2 entry in animal models. Sci. Rep..

[87] Khatri I., Staal F.J.T., van Dongen J.J.M. (2020). Blocking of the High-Affinity Interaction-Synapse Between SARS-CoV-2 Spike and Human ACE2 Proteins Likely Requires Multiple High-Affinity Antibodies: An Immune Perspective. Front. Immunol..

[88] Zhai S.-L., Wei W.-K., Lv D.-H., Xu Z.-H., Chen Q.-L., Sun M.-F., Li F., Wang D. (2020). Where did SARS-CoV-2 come from?. Vet. Rec..

[89] Lean F.Z.X., Núñez A., Spiro S., Priestnall S.L., Vreman S., Bailey D., James J., Wrigglesworth E., Suarez-Bonnet A., Conceicao C. (2022). Differential susceptibility of SARS-CoV-2 in animals: Evidence of ACE2 host receptor distribution in companion animals, livestock and wildlife by immunohistochemical characterisation. Transbound. Emerg. Dis..

[90] Chen D., Sun J., Zhu J., Ding X., Lan T., Zhu L., Xiang R., Ding P., Wang H., Wang X. (2020). Single-cell screening of SARS-CoV-2 target cells in pets, livestock, poultry and wildlife. bioRxiv.

[91] Le Poder S. (2011). Feline and canine coronaviruses: Common genetic and pathobiological features. Adv. Virol..

[92] Stavisky J., Pinchbeck G.L., German A.J., Dawson S., Gaskell R.M., Ryvar R., Radford A.D. (2010). Prevalence of canine enteric coronavirus in a cross-sectional survey of dogs presenting at veterinary practices. Vet. Microbiol..

[93] Andersen K.G., Rambaut A., Lipkin W.I., Holmes E.C., Garry R.F. (2020). The proximal origin of SARS-CoV-2. Nat. Med..

[94] Leroy E.M., Ar Gouilh M., Brugère-Picoux J. (2020). The risk of SARS-CoV-2 transmission to pets and other wild and domestic animals strongly mandates a one-health strategy to control the COVID-19 pandemic. One Health.

[95] Wan Y., Shang J., Graham R., Baric R.S., Li F. (2020). Receptor Recognition by the Novel Coronavirus from Wuhan: An Analysis Based on Decade-Long Structural Studies of SARS Coronavirus. J. Virol..

[96] Chen J., Huang C., Zhang Y., Zhang S., Jin M. (2020). Severe Acute Respiratory Syndrome Coronavirus 2-Specific Antibodies in Pets in Wuhan, China. J. Infect..

[97] Sailleau C., Dumarest M., Vanhomwegen J., Delaplace M., Caro V., Kwasiborski A., Hourdel V., Chevaillier P., Barbarino A., Comtet L. (2020). First detection and genome sequencing of SARS-CoV-2 in an infected cat in France. Transbound. Emerg. Dis..

[98] Fritz M., Rosolen B., Krafft E., Becquart P., Elguero E., Vratskikh O., Denolly S., Boson B., Vanhomwegen J., Gouilh M.A. (2021). High prevalence of SARS-CoV-2 antibodies in pets from COVID-19+ households. One Health.

[99] Hamer S.A., Pauvolid-Corrêa A., Zecca I.B., Davila E., Auckland L.D., Roundy C.M., Tang W., Torchetti M., Killian M.L., Jenkins-Moore M. (2020). Natural SARS-CoV-2 infections, including virus isolation, among serially tested cats and dogs in households with confirmed human COVID-19 cases in Texas, USA. bioRxiv.

[100] Patterson E.I., Elia G., Grassi A., Giordano A., Desario C., Medardo M., Smith S.L., Anderson E.R., Prince T., Patterson G.T. (2020). Evidence of exposure to SARS-CoV-2 in cats and dogs from households in Italy. Nat. Commun..

[101] Zambrano-Mila M.S., Freire-Paspuel B., Orlando S.A., Garcia-Bereguiain M.A. (2022). SARS-CoV-2 infection in free roaming dogs from the Amazonian jungle. One Health.

[102] Jairak W., Chamsai E., Udom K., Charoenkul K., Chaiyawong S., Techakriengkrai N., Tangwangvivat R., Suwannakarn K., Amonsin A. (2022). SARS-CoV-2 delta variant infection in domestic dogs and cats, Thailand. Sci. Rep..

[103] Zhao Y., Yang Y., Gao J., Huang K., Hu C., Hui X., He X., Li C., Gong W., Lv C. (2021). A serological survey of severe acute respiratory syndrome coronavirus 2 in dogs in Wuhan. Transbound. Emerg. Dis..

[104] Alberto-Orlando S., Calderon J.L., Leon-Sosa A., Patiño L., Zambrano-Alvarado M.N., Pasquel-Villa L.D., Rugel-Gonzalez D.O., Flores D., Mera M.D., Valencia P. (2022). SARS-CoV-2 transmission from infected owner to household dogs and cats is associated to food sharing. Int. J. Infect. Dis..

[105] Deng J., Jin Y., Liu Y., Sun J., Hao L., Bai J., Huang T., Lin D., Jin Y., Tian K. (2020). Serological survey of SARS-CoV-2 for experimental, domestic, companion and wild animals excludes intermediate hosts of 35 different species of animals. Transbound. Emerg. Dis..

[106] Jairak W., Charoenkul K., Chamsai E., Udom K., Chaiyawong S., Bunpapong N., Boonyapisitsopa S., Tantilertcharoen R., Techakriengkrai N., Surachetpong S. (2022). First cases of SARS-CoV-2 infection in dogs and cats in Thailand. Transbound. Emerg. Dis..

[107] Goryoka G.W., Cossaboom C.M., Gharpure R., Dawson P., Tansey C., Rossow J., Mrotz V., Rooney J., Torchetti M., Loiacono C.M. (2021). One Health Investigation of SARS-CoV-2 Infection and Seropositivity among Pets in Households with Confirmed Human COVID-19 Cases-Utah and Wisconsin, 2020. Viruses.

[108] Stevanovic V., Tabain I., Vilibic-Cavlek T., Mauric Maljkovic M., Benvin I., Hruskar Z., Kovac S., Smit I., Miletic G., Hadina S. (2021). The Emergence of SARS-CoV-2 within the Dog Population in Croatia: Host Factors and Clinical Outcome. Viruses.

[109] Bessière P., Vergne T., Battini M., Brun J., Averso J., Joly E., Guérin J.-L., Cadiergues M.-C. (2022). SARS-CoV-2 Infection in Companion Animals: Prospective Serological Survey and Risk Factor Analysis in France. Viruses.

[110] Bosco-Lauth A.M., Porter S.M., Fox K.A., Wood M.E., Neubaum D., Quilici M. (2022). Experimental Infection of Brazilian Free-Tailed Bats (*Tadarida brasiliensis*) with Two Strains of SARS-CoV-2. Viruses.

[111] Lau S.K., Luk H.K., Wong A.C., Li K.S., Zhu L., He Z., Fung J., Chan T.T., Fung K.S., Woo P.C. (2020). Possible Bat Origin of Severe Acute Respiratory Syndrome Coronavirus 2. Emerg. Infect. Dis..

[112] Csiszar A., Jakab F., Valencak T.G., Lanszki Z., Tóth G.E., Kemenesi G., Tarantini S., Fazekas-Pongor V., Ungvari Z. (2020). Companion animals likely do not spread COVID-19 but may get infected themselves. GeroScience.

[113] Almendros A. (2020). Can companion animals become infected with Covid-19?. Vet. Rec..

[114] Mallapaty S. (2020). Coronavirus can infect cats—Dogs, not so much. Nature.

[115] Singla R., Mishra A., Joshi R., Jha S., Sharma A.R., Upadhyay S., Sarma P., Prakash A., Medhi B. (2020). Human animal interface of SARS-CoV-2 (COVID-19) transmission: A critical appraisal of scientific evidence. Vet. Res. Commun..

[116] CDC (2020). Center for Disease Control: Interim Guidance for Public Health Professionals Managing People With COVID-19 in Home Care and Isolation Who Have Pets or Other Animals.

[117] Epidemiology Working Group for NCIP Epidemic Response, Chinese Center for Disease Control and Prevention (2020). The Epidemiological Characteristics of an Outbreak of 2019 Novel Coronavirus Diseases (COVID-19) in China. Zhonghua Liu Xing Bing Xue Za Zhi.

[118] Wang Q., Zhang Y., Wu L., Niu S., Song C., Zhang Z., Lu G., Qiao C., Hu Y., Yuen K.-Y. (2020). Structural and Functional Basis of SARS-CoV-2 Entry by Using Human ACE2. Cell.

[119] Marconato L., Finotello R. (2020). Veterinary oncologists adapting to COVID-19 pandemic. Vet. Comp. Oncol..

[120] Decaro N., Martella V., Saif L.J., Buonavoglia C. (2020). COVID-19 from veterinary medicine and one health perspectives: What animal coronaviruses have taught us. Res. Vet. Sci..

[121] Aitken M.M. (2020). Ensuring animal welfare during Covid-19 pandemic. Vet. Rec..

[122] Halfmann P.J., Hatta M., Chiba S., Maemura T., Fan S., Takeda M., Kinoshita N., Hattori S.-I., Sakai-Tagawa Y., Iwatsuki-Horimoto K. (2020). Transmission of SARS-CoV-2 in Domestic Cats. N. Engl. J. Med..

[123] Bowen J., Bulbena A., Fatjó J. (2021). The Value of Companion Dogs as a Source of Social Support for Their Owners: Findings From a Pre-pandemic Representative Sample and a Convenience Sample Obtained During the COVID-19 Lockdown in Spain. Front. Psychiatry.

[124] Morgan L., Protopopova A., Birkler R.I.D., Itin-Shwartz B., Sutton G.A., Gamliel A., Yakobson B., Raz T. (2020). Human–dog relationships during the COVID-19 pandemic: Booming dog adoption during social isolation. Hum. Soc. Sci. Commun..

[125] Holland K.E., Owczarczak-Garstecka S.C., Anderson K.L., Casey R.A., Christley R.M., Harris L., McMillan K.M., Mead R., Murray J.K., Samet L. (2021). “More Attention than Usual”: A Thematic Analysis of Dog Ownership Experiences in the UK during the First COVID-19 Lockdown. Animals.

[126] AVMA (2020). 37. American Veterinary Medical Association. Testing Animals for SARS-CoV-2. https://www.avma.org/resources-tools/animal-health-and-welfare/covid-19/testing-animals-sars-cov-2.

[127] Woolley C.S.C., Handel I.G., Bronsvoort B.M., Schoenebeck J.J., Clements D.N. (2022). The Impact of the COVID-19 Pandemic on a Cohort of Labrador Retrievers in England.

[128] USDA (2020). USDA Statement on the Confirmation of COVID-19 in a Tiger in New York.

[129] El-Sayed A., Kamel M. (2020). Climatic changes and their role in emergence and re-emergence of diseases. Environ. Sci. Pollut. Res..

[130] El-Sayed A., Kamel M. (2020). Future threat from the past. Environ. Sci. Pollut Res..

[131] Gunter L.M., Gilchrist R.J., Blade E.M., Reed J.L., Isernia L.T., Barber R.T., Foster A.M., Feuerbacher E.N., Wynne C.D.L. (2022). Emergency Fostering of Dogs From Animal Shelters During the COVID-19 Pandemic: Shelter Practices, Foster Caregiver Engagement, and Dog Outcomes. Front. Vet. Sci..

[132] Reese L.A., Jacobs J., Gembarski J., Opsommer C., Walker B. (2022). The COVID-19 Animal Fostering Boom: Ephemera or Chimera?. Animal.

[133] Vučinić M., Vučićević M., Nenadović K. (2022). The COVID-19 pandemic affects owners walking with their dogs. J. Vet. Behav..

[134] Harvey N.D., Christley R.M., Giragosian K., Mead R., Murray J.K., Samet L., Upjohn M.M., Casey R.A. (2022). Impact of Changes in Time Left Alone on Separation-Related Behaviour in UK Pet Dogs. Animal.

[135] OIE, World Organization for Animal Health (2020). Questions and Answers on the 2019 Coronavirus Disease (COVID-19). https://www.oie.int/scientific-expertise/specific-information-and-recommendations/questions-and-answers-on-2019novel-coronavirus/.

[136] Oliva J.L., Johnston K.L. (2021). Puppy love in the time of Corona: Dog ownership protects against loneliness for those living alone during the COVID-19 lockdown. Int. J. Soc. Psychiatry.

[137] Bussolari C., Currin-McCulloch J., Packman W., Kogan L., Erdman P. (2021). “I Couldn’t Have Asked for a Better Quarantine Partner!”: Experiences with Companion Dogs during Covid-19. Animals.

[138] Carr D., Friedmann E., Gee N.R., Gilchrist C., Sachs-Ericsson N., Koodaly L. (2021). Dog Walking and the Social Impact of the COVID-19 Pandemic on Loneliness in Older Adults. Animals.

[139] Mueller M.K., Richer A.M., Callina K.S., Charmaraman L. (2021). Companion Animal Relationships and Adolescent Loneliness during COVID-19. Animals.

[140] Kogan L.R., Erdman P., Bussolari C., Currin-McCulloch J., Packman W. (2021). The Initial Months of COVID-19: Dog Owners’ Veterinary-Related Concerns. Front. Vet. Sci..

[141] Shorunke F.O., Okolocha E.C., Kia G.S., Usman A., Akano O., Awosanya E.J. (2022). Prevalence and risk factors associated with SARS-CoV-2 infections among veterinary practitioners and dogs patients, June–August 2020, Lagos, Nigeria. One Health Outlook.

[142] Ga E., Won Y., Hwang J., Moon S., Yeom M., Lyoo K., Song D., Han J., Na W. (2022). A COVID-19 Vaccine for Dogs Prevents Reverse Zoonosis. Vaccines.

[143] Jepsen-Grant K., Pollard R.E., Johnson L.R. (2013). Vertebral heart scores in eight dog breeds. Vet. Radiol. Ultrasound.

[144] Romito G., Bertaglia T., Bertaglia L., Decaro N., Uva A., Rugna G., Moreno A., Vincifori G., Dondi F., Diana A. (2021). Myocardial Injury Complicated by Systolic Dysfunction in a COVID-19-Positive Dog. Animals.

[145] Recchia F.A., Lionetti V. (2007). Animal models of dilated cardiomyopathy for translational research. Vet. Res. Commun..

[146] Paradies P., Carlucci L., Woitek F., Staffieri F., Lacitignola L., Ceci L., Romano D., Sasanelli M., Zentilin L., Giacca M. (2019). Intracoronary Gene Delivery of the Cytoprotective Factor Vascular Endothelial Growth Factor-B 167 in Canine Patients with Dilated Cardiomyopathy: A Short-Term Feasibility Study. Vet. Sci..

[147] Colitti B., Manassero L., Colombino E., Ferraris E.I., Caccamo R., Bertolotti L., Bortolami A., Bonfante F., Papa V., Cenacchi G. (2022). Pulmonary fibrosis in a dog as a sequela of infection with Severe Acute Respiratory Syndrome Coronavirus 2? A case report. BMC Vet. Res..

[148] Wiersinga W.J., Rhodes A., Cheng A.C., Peacock S.J., Prescott H.C. (2020). Pathophysiology, Transmission, Diagnosis, and Treatment of Coronavirus Disease 2019 (COVID-19). JAMA.

[149] Eskandari E., Ahmadi Marzaleh M., Roudgari H., Hamidi Farahani R., Nezami-Asl A., Laripour R., Aliyazdi H., Dabbagh Moghaddam A., Zibaseresht R., Akbarialiabad H. (2021). Sniffer dogs as a screening/diagnostic tool for COVID-19: A proof of concept study. BMC Infect. Dis..

[150] Sakr R., Ghsoub C., Rbeiz C., Lattouf V., Riachy R., Haddad C., Zoghbi M. (2021). COVID-19 detection by dogs: From physiology to field application-a review article. Postgrad. Med. J..

[151] McCulloch M., Jezierski T., Broffman M., Hubbard A., Turner K., Janecki T. (2006). Diagnostic Accuracy of Canine Scent Detection in Early- and Late-Stage Lung and Breast Cancers. Integr. Cancer Ther..

[152] Taylor M.T., McCready J., Broukhanski G., Kirpalaney S., Lutz H., Powis J. (2018). Using Dog Scent Detection as a Point-of-Care Tool to Identify Toxigenic Clostridium difficile in Stool. Open Forum Infect. Dis..

[153] Angle C., Waggoner L.P., Ferrando A., Haney P., Passler T. (2016). Canine Detection of the Volatilome: A Review of Implications for Pathogen and Disease Detection. Front. Vet. Sci..

[154] Angle T.C., Passler T., Waggoner P.L., Fischer T.D., Rogers B., Galik P.K., Maxwell H.S. (2016). Real-Time Detection of a Virus Using Detection Dogs. Front. Vet. Sci..

[155] Koivusalo M., Reeve C. (2018). Biomedical scent detection dogs: Would they pass as a health technology?. Pet. Behav. Sci.

[156] Amann A., Costello B.d.L., Miekisch W., Schubert J., Buszewski B., Pleil J., Ratcliffe N., Risby T. (2014). The human volatilome: Volatile organic compounds (VOCs) in exhaled breath, skin emanations, urine, feces and saliva. J. Breath Res..

[157] Shirasu M., Touhara K. (2011). The scent of disease: Volatile organic compounds of the human body related to disease and disorder. J. Biochem..

[158] Jendrny P., Schulz C., Twele F., Meller S., Von Köckritz-Blickwede M., Osterhaus A.D.M.E., Ebbers J., Pilchová V., Pink I., Welte T. (2020). Scent dog identification of samples from COVID-19 patients—A pilot study. BMC Infect. Dis.

[159] Grandjean D., Sarkis R., Lecoq-Julien C., Benard A., Roger V., Levesque E., Bernes-Luciani E., Maestracci B., Morvan P., Gully E. (2020). Can the detection dog alert on COVID-19 positive persons by sniffing axillary sweat samples? A proof-of-concept study. PLoS ONE.

[160] Angeletti S., Travaglino F., Spoto S., Pascarella M.C., Mansi G., de Cesaris M., Sartea S., Giovanetti M., Fogolari M., Plescia D. (2021). COVID-19 Sniffer Dog experimental training: Which protocol and which implications for reliable identification?. J. Med. Virol..

[161] Grandjean D., Al Marzooqi D.H., Lecoq-Julien C., Muzzin Q., Al Hammadi H.K., Alvergnat G., Al Blooshi K.M., Al Mazrouei S.k., Alhmoudi M.S., Al Ahbabi F.M. (2021). Use of Canine Olfactory Detection for COVID-19 Testing Study on U.A.E. Trained Detection Dog Sensitivity. bioRxiv.

[162] Sharun K., Jose B., Tiwari R., Natesan S., Dhama K. (2021). Biodetection dogs for COVID-19: An alternative diagnostic screening strategy. Public Health.

[163] Sarkis R., Lichaa A., Mjaess G., Saliba M., Selman C., Lecoq-Julien C., Grandjean D., Jabbour N.M. (2021). New method of screening for COVID-19 disease using sniffer dogs and scents from axillary sweat samples. J. Public Health.

[164] Hag-Ali M., AlShamsi A.S., Boeijen L., Mahmmod Y., Manzoor R., Rutten H., Mweu M.M., El-Tholoth M., AlShamsi A.A. (2021). The detection dogs test is more sensitive than real-time PCR in screening for SARS-CoV-2. Commun. Biol..

[165] Maurer M., Seto T., Guest C., Somal A., Julian C. (2022). Detection of SARS-CoV-2 by Canine Olfaction: A Pilot Study. Open Forum Infect. Dis..

[166] Mancilla-Tapia J.M., Lozano-Esparza V., Orduña A., Osuna-Chávez R.F., Robles-Zepeda R.E., Maldonado-Cabrera B., Bejar-Cornejo J.R., Ruiz-León I., González-Becuar C.G., Hielm-Björkman A. (2022). Dogs Detecting COVID-19 From Sweat and Saliva of Positive People: A Field Experience in Mexico. Front. Med..

[167] Grandjean D., SLAMA D., Gallet C., Julien C., SEYRAT E., BLONDOT M., BENAZAZIEZ M., ELBAZ J., SALMON D. (2022). Screening for SARS-CoV-2 Persistence in Long COVID Patients Using Sniffer Dogs and Scents from Axillary Sweats Samples.

[168] Grandjean D., Gallet C., Julien C., Sarkis R., Muzzin Q., Roger V., Roisse D., Dirn N., Levert C., Breton E. (2022). Identifying SARS-COV-2 infected patients through canine olfactive detection on axillary sweat samples; study of observed sensitivities and specificities within a group of trained dogs. PLoS ONE.

[169] Kantele A., Paajanen J., Turunen S., Pakkanen S.H., Patjas A., Itkonen L., Heiskanen E., Lappalainen M., Desquilbet L., Vapalahti O. (2022). Scent dogs in detection of COVID-19: Triple-blinded randomised trial and operational real-life screening in airport setting. BMJ Glob. Health.

[170] Grandjean D., Elie C., Gallet C., Julien C., Roger V., Desquilbet L., Alvergnat G., Delarue S., Gabassi A., Minier M. (2022). Diagnostic Accuracy of Non-Invasive Detection of SARS-CoV-2 Infection by Canine Olfaction.

[171] Dickey T., Junqueira H. (2021). Toward the use of medical scent detection dogs for COVID-19 screening. J. Osteopath. Med..

[172] Vesga O., Agudelo M., Valencia-Jaramillo A.F., Mira-Montoya A., Ossa-Ospina F., Ocampo E., Čiuoderis K., Pérez L., Cardona A., Aguilar Y. (2021). Highly sensitive scent-detection of COVID-19 patients in vivo by trained dogs. PLoS ONE.

[173] Otto C.M., Sell T.K., Veenema T.G., Hosangadi D., Vahey R.A., Connell N.D., Privor-Dumm L. (2021). The Promise of Disease Detection Dogs in Pandemic Response: Lessons Learned From COVID-19. Disaster Med. Public Health Prep..

[174] Devillier P., Gallet C., Salvator H., Lecoq-Julien C., Naline E., Roisse D., Levert C., Breton E., Galtat A., Decourtray S. (2022). Biomedical detection dogs for the identification of SARS-CoV-2 infections from axillary sweat and breath samples. J. Breath Res..

[175] Tetro J.A. (2020). Is COVID-19 receiving ADE from other coronaviruses?. Microbes Infect..

[176] Tilocca B., Soggiu A., Sanguinetti M., Babini G., de Maio F., Britti D., Zecconi A., Bonizzi L., Urbani A., Roncada P. (2020). Immunoinformatic analysis of the SARS-CoV-2 envelope protein as a strategy to assess cross-protection against COVID-19. Microbes Infect..

[177] Tilocca B., Soggiu A., Sanguinetti M., Musella V., Britti D., Bonizzi L., Urbani A., Roncada P. (2020). Comparative computational analysis of SARS-CoV-2 nucleocapsid protein epitopes in taxonomically related coronaviruses. Microbes Infect..

[178] Marinella M.A. (2020). Indomethacin and resveratrol as potential treatment adjuncts for SARS-CoV-2/COVID-19. Int. J. Clin. Pract..

